# Learning during COVID-19: the role of self-regulated learning, motivation, and procrastination for perceived competence

**DOI:** 10.1007/s11618-021-01002-x

**Published:** 2021-03-04

**Authors:** Elisabeth Rosa Pelikan, Marko Lüftenegger, Julia Holzer, Selma Korlat, Christiane Spiel, Barbara Schober

**Affiliations:** 1 Educational Psychology, Department of Developmental and Educational Psychology, Faculty of Psychology, University of Vienna, Universitätsstraße 7, 1010 Vienna, Austria; 2 Department for Teacher Education, Centre for Teacher Education, University of Vienna, Porzellangasse 4, 1090 Vienna, Austria

**Keywords:** COVID-19, Intrinsic motivation, Perceived competence, Procrastination, Self-regulated learning, COVID-19, Intrinsische Motivation, Prokrastination, Selbstreguliertes Lernen, Wahrgenommene Kompetenz

## Abstract

In March 2020 schools in Austria temporarily closed and switched to distance learning to contain the spread of the coronavirus (COVID-19). The resulting situation posed great challenges to teachers, guardians and students (Huber and Helm 2020). Research has shown that perceived competence (Deci and Ryan 2000) affects selfregulated learning (SRL), intrinsic motivation and procrastination, however few studies have considered these variables in context of distance learning among adolescents. This study investigated differences in students who perceived themselves as high vs. low in competence with respect to these constructs. In an online questionnaire, 2652 Austrian secondary school students answered closed questions regarding SRL, intrinsic motivation and procrastination as well as open-ended questions about challenges, successes and need for support in distance. Structural equation modeling was applied for the quantitative analysis which was complemented by thematic analysis for the qualitative questions (Braun and Clarke 2006). Results showed that students who experienced themselves as highly competent use SRL strategies (goal setting and planning, time management, metacognitive strategies) more often and are more intrinsically motivated than students with lower perceived competence. They also procrastinate less. Furthermore, qualitative analysis revealed that although all students face similar challenges (e.g., independent learning, time and task management, learning on the computer, lack of contact with teachers and peers), students who perceived themselves as highly competent seemed to cope better, and have less need for support. Implications for distance learning and future research are discussed.

**Supplementary Information:**

The online version of this article (10.1007/s11618-021-01002-x) contains supplementary material, which is available to authorized users.

## Abstract (de)

Im März 2020 wurden in Österreich Schulen vorübergehend geschlossen und auf Lernen auf Distanz umgestellt, um die Ausbreitung des Coronavirus (COVID-19) einzudämmen. Die daraus resultierende Situation stellte Lehrer, Erziehungsberechtigte und Schüler*innen vor große Herausforderungen (Huber und Helm 2020). Obwohl bisherige Forschung gezeigt hat, dass wahrgenommene Kompetenz selbstreguliertes Lernen (SRL), intrinsische Motivation und Prokrastination beeinflusst, haben sich nur wenige Studien mit diesen im Kontext des Lernens auf Distanz bei Jugendlichen befasst. Die vorliegende Studie untersuchte die Unterschiede zwischen Schüler*innen, die sich selbst als hoch vs. niedrig kompetent wahrnahmen und inwieweit diese Variablen hierfür eine Rolle spielen. In einem Online-Fragebogen beantworteten 2652 österreichische Schüler*innen der Sekundarstufe geschlossene Fragen zu SRL, intrinsischer Motivation und Prokrastination sowie offene Fragen zu Herausforderungen und Erfolgen beim Lernen auf Distanz und dem damit zusammenhängenden Unterstützungsbedarf. Für die quantitative Analyse wurde ein Strukturgleichungsmodell berechnet, welches durch eine thematische Analyse der qualitativen Fragen ergänzt wurde (Braun und Clarke 2006). Die Ergebnisse zeigten, dass Schüler*innen, die sich selbst als hochkompetent erleben, häufiger SRLStrategien (Zielsetzung und Planung, Zeitmanagement, metakognitive Strategien) anwenden und höhere intrinsische Motivation aufweisen, als Schüler*innen mit geringer wahrgenommener Kompetenz. Sie prokrastinieren außerdem weniger. Darüber hinaus ergab die qualitative Analyse, dass, obwohl alle Schüler*innen mit ähnlichen Herausforderungen konfrontiert sind (z. B. selbständiges Lernen, Zeit- und Aufgabenmanagement, Lernen am Computer, mangelnder Kontakt mit Lehrern und Gleichaltrigen), jene Schüler*innen, die sich selbst als hochkompetent wahrnehmen, besser damit zurechtkommen und weniger Unterstützung benötigen. Implikationen für das Lernen auf Distanz und zukünftige Forschung werden diskutiert.

In March 2020, the novel coronavirus (COVID-19) was classified as a pandemic by the World Health Organization (WHO 2020). In addition to other social distancing measures (e.g., curfews and business shutdowns), many countries temporarily closed schools and switched to distance learning to contain the spread of the virus (UNICEF 2020). The resulting situation posed great challenges for all actors in the educational context. Teachers had to develop new concepts for “emergency teaching at a distance” (Bozkurt et al. 2020) to ensure that lessons could continue without disruption. Parents partially took over on the role of teachers in addition to their work and household obligations (Huber et al. 2020; Viner et al. 2020). Students also found themselves in a novel situation. While in face-to-face teaching, fixed structures regulated daily school life and learning time, students now had to organize and self-regulate their learning autonomously with little time for preparation from one day to the next. Self-regulated learning (SRL; planning, monitoring and adapting one’s thoughts, feelings and actions in a cyclical process to attain a personal goal; Zimmerman 2000) and intrinsic motivation are considered important factors for learning success in face-to-face settings (Dent and Koenka 2016; Fortier et al. 1995; Zimmerman 1990), and gain additional relevance when students face a situation such as distance learning with less external structure and guidance (Dabbagh and Kitsantas 2004). Furthermore, intrinsic motivation, goal setting, and SRL are influenced by perceived competence, that is, by the learner’s feeling of being able to handle given tasks (e.g., Cho et al. 2011; Ferla et al. 2010; Miller et al. 1993). A lack of both intrinsic motivation and of self-regulation has been associated with higher passive procrastination (Steel 2007), which has in turn been associated with various detrimental behaviors and outcomes (Howell and Watson 2007; Steel 2007) in an academic context.

The aim of the current study was to investigate students differing in their perceived competence when experiencing learning under COVID-19 conditions. We compare students who perceive themselves as highly competent to those who perceive themselves as having low competence in this situation; specifically, we investigate how they differ in terms of variables that are considered decisive for successful learning in many studies: SRL, intrinsic learning motivation, and passive procrastination as well as their experiences of challenges and successes during learning.

## Theoretical background

### Distance learning during the COVID-19 crisis

Simonson and Berg (2016) define distance learning as a “form of education in which the main elements include physical separation of teachers and students during instruction and the use of various technologies to facilitate student-teacher and student-student communication”. Previous studies on distance learning conclude that it can be just as effective as face-to-face teaching (Cavanaugh et al. 2004; Lee and Figueroa 2012; Means et al. 2013). However, COVID-19 is a new and challenging situation with respect to distance learning, as mandatory school shutdowns due to a pandemic or natural catastrophe have never before been necessary at such a large scale (Huber and Helm 2020).

During the COVID-19 lockdown, in addition to switching to distance learning, most schools used digital platforms to support instruction during school closures (Huber and Helm 2020). Although the digitization of teaching and learning has frequently been called for in connection with the notion of lifelong learning and adaptation to the modern labor market (European Commission 2018), it had not yet been implemented across the board in Austria before COVID-19, especially in primary and secondary education (Schrenk 2020; Wahlmüller-Schiller 2017). It is therefore particularly important to investigate how students were able to cope with this new situation and how students who perceived themselves as competent differed from those who did not.

### Perceived competence, self-regulated learning, motivation and passive procrastination in distance learning

Distance learning in comparison to regular face-to-face lessons is characterized by greater flexibility in scheduling, the opportunity to individualize learning processes, the potential to enhance SRL skills and the easy distribution of information (Means et al. 2013; Mupinga 2005; Paechter and Maier 2010; Rice 2006). However, this can present both advantages and disadvantages, especially for younger students (Cavanaugh et al. 2004; Mupinga 2005), as the greater flexibility available in distance learning places high demands on the learner’s ability to regulate their learning and motivation (Adam et al. 2017; Dabbagh and Kitsantas 2004; Fryer and Bovee 2016; Fryer et al. 2014) and thus poses an increased risk of passive procrastination (Rakes and Dunn 2010).

SRL has long been recognized as an important contributor to learning success in traditional as well as online learning settings (Dent and Koenka 2016; Donker et al. 2014; Zimmerman 1990). Although various theoretical models have been proposed (see Panadero 2017 for an overview), common to all is that self-regulated learners are able to actively control their learning process by setting achievable goals, managing their time and tasks, monitoring their progress, regulating their motivation and seeking help when necessary (Zeidner et al. 2000). Distance learning is typically less structured and therefore relies on learners to autonomously regulate and organize their learning processes. Metacognitive strategies (e.g., monitoring and evaluating progress towards goal achievement, adjusting learning strategies if necessary, mobilizing personal and environmental resources) as well as other SRL strategies such as setting goals and managing one’s time are considered to be even more important in distance learning than in traditional learning settings (Dabbagh and Kitsantas 2004). Furthermore, several studies have indicated that the use of SRL strategies changes with age (Zimmerman and Martinez-Pons 1990; Cavanaugh et al. 2004), suggesting that younger students need more support in regulating their learning.

Another factor driving academic success is intrinsic motivation. While extrinsic motivation, which involves external rewards or punishments, may be detrimental to academic achievement and well-being (see, for example, Ryan and Deci 2000), intrinsic motivation has been proven to be an important predictor for learning success in the form of e.g., reading achievement (Froiland and Oros 2014) and higher grades (Fortier et al. 1995; Ning and Downing 2010). According to self-determination theory (SDT; Deci and Ryan 2000, 2008; Ryan and Deci 2000), intrinsic motivation arises if an activity satisfies the need for perceived competence and autonomy (supported by social relatedness). Perceived competence refers to an individual “feeling competent with tasks and activities” (Chen and Jang 2010, p. 742), while autonomy refers to a feeling of agency (Deci and Ryan 1985; Reeve et al. 2003). In the educational context, students who feel that they have choice and responsibility in their learning (*autonomy*) and feel competent in mastering their tasks (*competence*) should experience increased intrinsic motivation (Deci and Ryan 2000). However, while autonomous learning situations such as distance learning can increase motivation, they do so only if actors perceive themselves as competent and able to handle the associated challenges and achieve their goals (Deci and Ryan 2000). Therefore, perceived competence acts as an important predictor of intrinsic motivation (Cho et al. 2011; Guay et al. 2001; Stephan et al. 2011; Zisimopoulos and Galanaki 2009). Results of previous studies have shown that perceived competence also influences the use of SRL strategies such as goal setting, monitoring and the use of metacognitive strategies (Miller et al. 1993; Pichardo et al. 2014; Zimmerman and Martinez-Pons 1986). Studies have also found that intrinsic motivation may vary with age (Gillet et al. 2012; Gottfried et al. 2001).

Whereas the use of SRL strategies and high motivation have been shown to predict academic achievement, passive procrastination is usually associated with various kinds of detrimental behaviors and outcomes. Passive procrastination in the academic context means delaying school-related tasks even when faced with negative consequences (Steel 2007; Steel and Klingsieck 2016). Notably, passive procrastination has been differentiated from active procrastination, which describes an intentional and strategic delay of tasks and is seen as an act of self-regulation and not associated with negative consequences (Steel 2007). Passive procrastination has been associated with lower goal commitment and less use of organizational as well as metacognitive learning strategies (Howell and Watson 2007; Steel 2007). It has also been shown to correlate with lower academic outcomes (e.g., lower GPA; Steel 2007). Ryan and Deci (2000) suggest that passive procrastination is simply the opposite of motivation. Wolters (2003), however, found that the use of metacognitive SRL strategies also plays a role in the tendency to passively procrastinate. This finding was echoed by Steel (2007) in his meta-analysis, which found that low self-control, low self-discipline and organization and low achievement motivation are all strong predictors of passive procrastination. In contrast to SRL and motivation, the role of perceived competence for passive procrastination is less often examined. Perceived competence was found to be a negative predictor of students’ passive procrastination on academic tasks (Brando-Garrido et al. 2020). Another study found that the link between fear of failure and passive procrastination was moderated by perceived competence, indicating students with higher perceived competence could handle challenges more positively than students with low perceived competence (Haghbin et al. 2012).

Despite several studies documenting the importance of perceived competence for SRL and motivation and the increased risk of passive procrastination in distance learning (e.g., Ferla et al. 2010), to our knowledge, the interplay between these constructs has not been investigated so far. The current study aims to fill this gap by focusing on the role of perceived competence in SRL and motivation during the COVID-19 pandemic, which provides a unique opportunity to investigate the interplay among these constructs in distance learning.

### The present study

Distance learning requires high self-regulation and intrinsic motivation and carries the risk of passive procrastination. Therefore, SRL skills became imperative when switching to distance learning during the COVID-19 pandemic. Previous research indicates that perceived competence is related to various aspects of self-regulated learning, such as goal setting, time management and the use of metacognitive strategies. Furthermore, autonomous learning situations such as distance learning may improve intrinsic motivation, but only in connection with high perceived competence.

Therefore, students who perceive themselves as highly competent should differ in their use of self-regulated learning strategies, their intrinsic motivation and passive procrastination from students who perceive themselves as less competent. In our study, we separated students based on their self-reported perceived competence, drawing from a larger sample of around 19,000 students. This allowed us to build true extreme groups of still considerable sample size.

Furthermore, there is a lack of deeper knowledge about the different mechanisms used by students who perceive themselves as highly competent in contrast to students who perceive themselves as lacking competence in new and challenging learning situations, such as distance learning during COVID-19. In our study, we complimented our quantitative scales with qualitative data and asked students about their challenges, successes and need for support in an open-ended format.

Our first research question addresses the differences between students who perceive themselves as highly competent and students who perceive themselves as lacking competence in important aspects of self-regulated learning (time management, goals and plans, metacognition), intrinsic learning motivation and procrastination. In line with previous studies, we expect students who perceive themselves as highly competent to report higher scores on all SRL aspects (Zimmerman and Martinez-Pons 1986) and on intrinsic motivation (Fortier et al. 1995; Froiland and Oros 2014; Ning and Downing 2010; Rakes and Dunn 2010; Wang et al. 2013, 2019) as well as lower passive procrastination scores (Brando-Garrido et al. 2020; Haghbin et al. 2012) than students who perceive themselves as lacking competence.

In our second research question, we were interested in the mechanisms applied by students who perceived themselves as high in vs. lacking competence in mastering the distance learning situation. We asked students about their experiences in terms of challenges as well as positive aspects and need for support. Applying thematic analysis (Braun and Clarke 2006), we again focused on differences between students who perceived themselves as highly vs. lacking competence.

## Methods

### Participants, procedure and context of data collection

This study was part of a larger study on learning during COVID-19 (Schober et al. 2020). The full sample comprised 19,337 secondary school students (37.9% males, 61.6% females, 0.5% diverse) with a mean age of 14.56 years (*SD*_age_ = 2.49, Mdn = 14.00, Range = 10–21). Data were collected with online questionnaires from April 7th to April 24th, 2020. To recruit the sample, we distributed the link to the online questionnaire by contacting manifold stakeholders such as school boards, educational networks, and school principals. Additionally, the Austrian Federal Ministry for Education, Science, and Research recommended participation in the study and published the link on its website. We also received support from several media outlets. Participants were informed about the study’s goals, inclusion criteria for participation, i.e., attending secondary school in Austria, and the complete anonymity of their data. All students participated voluntarily and only those who gave active consent were included in the dataset. In Austria, schools stopped providing onsite learning on March 16th. Also, as of March 16th, the government announced that homes could only be left for work, making necessary purchases, assisting other people or outdoor activities, alone or in the company of people living in the same household. During the full data collection period, schools were obliged to ensure continued education by providing distance learning. Teachers and schools were autonomous in the organization and design of remote teaching. Although there was no on-site teaching, schools remained open, as they provided childcare in necessary cases (Federal Ministry of Education 2020b). However, this offer was only taken up by roughly 2% of the student population (Federal Ministry of Education 2020a).

### Sample selection

For this study, a subsample of students was identified from the larger survey study. Using an adapted version of the Work-related Basic Need Satisfaction Scale (W-BNS; Van den Broeck et al. 2010) with three items, which were modified to suit the school context (sample item: “These days I am able to successfully complete most of my schoolwork”; CR = 0.85 for the whole sample), all students with scale means of = 1 (low perceived competence; *n* = 235) and = 5 (high perceived competence, *n* = 2417) were selected to build extreme groups (low and high perceived competence, respectively). Therefore, the sample for this study consisted of 2652 Austrian secondary school students (37.6% males, 61.9% females, 0.6% diverse) with a mean age of 14.15 years (*SD*_age_ = 2.53, Mdn = 14.00, Range = 10–21). The students stemmed from secondary schools all over Austria: academic-track secondary schools (“Gymnasium”; 31.2%), comprehensive middle schools (“Neue Mittelschule”, “Mittelschule”, “Hauptschule”, “Polytechnische Schule”, or “Fachmittelschule”; 39.4%), academic-track vocational schools (“Berufsbildende Höhere Schule”; 24.6%), lower-track vocational schools and dual vocational education (“Berufsbildende Mittlere Schule” or “Berufsschule und Lehre”; 4.2%), and other school types, including inclusive education (0.6%).

### Measures

Due to the novelty of the COVID-19 situation, it was necessary to adapt existing scales or newly develop items to fit the current circumstances. To ensure the construct validity of the measures, we conducted confirmatory factor analyses (CFA) and analyzed composite reliability (CR; Raykov 2009). All items were rated on a 5-point scale ranging from 1 (strongly agree) to 5 (strongly disagree). Participants were instructed to answer the items with respect to the current situation (learning from home due to the coronavirus). We conducted analyses with recoded items so that higher values reflected higher agreement with the statements. We used three scales (*Goals and plans, Time, Metacognition*) to measure aspects of SRL.

*Goals and plans *in terms of setting goals and planning one’s learning process was assessed with three items, slightly adapted from the short version of the Learning Strategies of University Students questionnaire (LIST‑K; Klingsieck 2018; sample item: “When I am currently studying, I make a plan of what I need to do”; CR = 0.80).

*Time *was assessed with three items also adapted from the LIST‑K (Klingsieck 2018; sample item: “When I am currently studying, I reserve specific times for studying each day”; CR = 0.72).

*Metacognition *was measured with three items. The first item was newly developed for the questionnaire (“When I am currently studying, I try to motivate myself (e.g., through rewards for each completed task)”), while the other two were adapted from the LIST‑K (Klingsieck 2018; “When I am currently studying, I try out different ways when something is not working”; “When I am currently studying, I seek out feedback when I need it”; CR = 0.62). The three items cover three important aspects of metacognition: motivation regulation, monitoring, and resource management.

*Passive procrastination *was measured with three items slightly adapted from the Procrastination Questionnaire for Students (PFS; Glöckner-Rist et al. 2014; sample item: “I put off tasks until the last minute”; CR = 0.86).

*Intrinsic learning motivation* was assessed with three items adapted from the Scales for the Measurement of Motivational Regulation for Learning in University Students (SMR-LS; Thomas et al. 2018; sample item: “Currently, I really enjoy studying and working for school”; CR = 0.93).

In addition to the quantitative questions, the survey contained six open-ended questions, three of which were used in this study. In Question 1, students were asked about challenges regarding distance learning during the COVID-19 crisis (“What do you currently find especially hard when studying?”). Question 2 asked whether students had success in distance learning (“What parts of studying are currently going particularly well?”) and Question 3 addressed students’ need for support (“With what could you currently use some help?”).

### Data analysis

#### Quantitative analysis.

Quantitative data were analyzed using SPSS version 25.0 (IBM 2017) and Mplus version 8.4 (Muthén and Muthén 2017). To deal with the very small number of missing values (ranging from 0.0% to 0.6% on the item level), the full information maximum likelihood approach implemented in Mplus was employed. All statistical significance testing for the quantitative analyses were performed at the 0.05 level. However, due to the large sample, rather than relying on statistical significance, we particularly focused on the identified effect sizes when interpreting the obtained results. To interpret the effect sizes of regression parameters, we followed Cohen (1988), according to whom standardized values of 0.10, 0.30, and 0.50 reflect small, moderate, and large effect sizes, respectively.

First, confirmatory factor analyses using robust maximum likelihood estimation (MLR) were conducted to analyze the scales’ construct validity. Goodness-of-fit was evaluated using the χ^2^ Test of Model Fit, Tucker-Lewis index (TLI), Comparative Fit Index (CFI), root mean square error of approximation (RMSEA) and Standardized Root Mean Residual (SRMR). In addition, a 90% confidence interval around the point allowed us to estimate the precision of the RMSEA estimate. We considered typical cutoff scores reflecting excellent and adequate fit to the data, respectively: (a) CFI and TLI > 0.95 and 0.90; (b) RMSEA < 0.06 and 0.08; (c) SRMR < 0.08 (Hu and Bentler 1999). Additionally, we relied on the comparative model fit indices AIC and BIC for model comparison, with lower values indicating a better trade-off between fit and complexity. We tested three CFA models for the five investigated constructs. In our first model, the items loaded onto the five different constructs as expected (*Goals and plans, Time, Meta-cognition, Intrinsic learning motivation, Passive procrastination*). All standardized factor loadings were moderate to strong (ranging from 0.49 to 0.94) and the model demonstrated excellent model fit indices, χ^2^ (80) = 566.12, *p* < 0.001, CFI = 0.97, TLI = 0.96, RMSEA = 0.048 [0.044, 0.052], SRMR = 0.039, AIC = 112,082.92, BIC = 112,406.41. We additionally tested the hypothesized model against a one-factor model, χ^2^ (90) = 5880.70, *p* < 0.001, CFI = 0.59, TLI = 0.53, RMSEA = 0.156 [0.153, 0.159], SRMR = 0.117, AIC = 118,985.53, BIC = 119,250.20, and a model with a SRL (*Goals and plans, Time, Meta-cognition*) and a motivation factor (*Intrinsic learning motivation, Passive procrastination*), χ^2^ (84) = 950.43, *p* < 0.001, CFI = 0.94, TLI = 0.92, RMSEA = 0.062 [0.059, 0.066], SRMR = 0.047, AIC = 112,571.57, BIC = 112,871.53. Our hypothesized model showed the best model fit.

Second, we set up a SEM using the MLR estimator to test main effects (*Goal and plans, Time, Metacognition, Passive procrastination, Intrinsic learning motivation*) between the two groups. We conducted CFAs with covariates for all five outcome variables. We introduced group (0 = low perceived competence; 1 = high perceived competence) as a predictor for the outcome variables and additionally we controlled for age. We report standardized coefficients (b*) and standard errors (*SE*).

#### Qualitative analysis.

Qualitative data analysis of the open-ended questions was conducted by applying thematic analysis (Braun and Clarke 2006), using the software MAXQDA 2020 (VERBI Software 2019) to support the coding process. The categories were generated based on a mixed deductive and inductive approach. First, 300 answers to each question were screened separately by the first author to familiarize herself with the data. Initial codes were generated based on current research on distance learning as presented in the literature review and applied to the data (deductive approach). Subsequently, further main themes and subthemes based on the data were identified and added to the category system (inductive approach). After this procedure, the categories were defined and arranged in a systematic order. This step also included the formulation of category descriptions with inclusion and exclusion criteria as well as examples (see Tables IV–VI in the supplementary material for the final categorization system for each question). After this systematic coding of 300 answers, the coding system for each question was reviewed, which involved redefining and rearranging the themes and subthemes. Afterwards, a random sample of 25% of the data for each question were coded separately by the first author and a second trained researcher. Interrater reliability checks were conducted and found to be satisfactory, with Cohen’s Kappa ranging from *κ* = 0.89 to *κ* = 0.95. Disagreements were discussed, and category descriptions as well as the order of categories, were further refined. Finally, the first author completed the coding for the remaining data.

To test for differences in the number of coded segments in the high, respectively low perceived competence group, chi-squared tests for the number of coded segments were computed for each of the main categories and subcategories. As conducting a large number of significance tests bears the risk of alpha error accumulation, significance testing was performed at the 0.01 level.

## Results

Table 1 provides bivariate latent correlations among all variables as well as descriptive statistics and composite reliabilities.

**Table 1:** Bivariate Latent Correlations, Descriptive Statistics and Composite Reliabilities

	1	2	3	4	5
1. Goals and Plans	–				
2. Time	0.73	–			
3. Meta-Cognition	0.67	0.63	–		
4. Procrastination	−0.48	−0.40	−0.40	–	
5. Intrinsic learning motivation	0.53	0.47	0.62	−0.47	–
Number of Items	3	3	3	3	3
*M*	4.42	3.37	3.87	1.88	3.71
*SD*	0.89	1.16	0.99	1.12	1.23
Skewness	−1.99	−0.41	−0.84	1.44	−0.91
Kurtosis	3.80	−0.77	0.10	1.16	−0.16
Range	4.00	4.00	4.00	4.00	4.00
Composite Reliability	0.80	0.72	0.62	0.86	0.93

*Note. N* = 2652 students*. *All scales used a 5-point response format. *CR* Composite Reliability. All correlation coefficients are statistically significant at *p* < 0.001

### Main effects of intrinsic learning motivation, passive procrastination and self-Regulated learning.

In order to investigate whether there are differences between students who perceived themselves as high in vs. lacking competence in terms of intrinsic learning motivation, passive procrastination and self-regulated learning, CFAs with covariates were conducted. The effect of age on the outcome variables of passive procrastination and time was controlled for (both variables showed small age effects with r > |0.1|). Overall fit indices indicated an excellent model fit, χ^2^ (103) = 803.376, *p* < 0.001, CFI = 0.95, TLI = 0.93, RMSEA = 0.051 [0.047, 0.054], SRMR = 0.041. Estimation revealed that group (low vs. high perceived competence) positively predicted students’ goals and plans, b* = 0.47, SE = 0.06, *p* < 0.001, time, b* = 0.37, SE = 0.05, *p* < 0.001, metacognition, b* = 0.51, SE = 0.07, *p* < 0.001, and intrinsic learning motivation, b* = 0.59, SE = 0.06, *p* < 0.001, with students with high perceived competence expressing higher levels of the outcome variables. Moreover, passive procrastination was negatively predicted by group, b* = −0.46, SE = 0.05, *p* < 0.001, indicating that students with high perceived competence exhibited less passive procrastination than students with low perceived competence.

### Challenges, successes and need for support in distance learning

In the thematic analysis, five main themes emerged, reoccurring in all three open-ended questions, but differing slightly in their individual subcategories. These main themes were *Contact with others* (family/parents or guardians, peers and teachers),* Learning outcomes, Learning process, Contextual conditions *and* Well-being*. The category systems for all three questions were developed based on these main themes. In the following section, narrative summaries of the main categories are provided separately for each question. The absolute and relative numbers of coded segments for the total sample as well as for each group and the according Chi-square tests can be found in the supplementary material. Since only *n* = 235 answers were available from the low perceived competence group (compared to *n* = 2417 answers in the high perceived competence group), the number of coded segments was set in relation to the number of segments coded in each group rather than to the overall number of coded segments in the full sample.

#### Challenges in distance learning

**Fig. 1:**
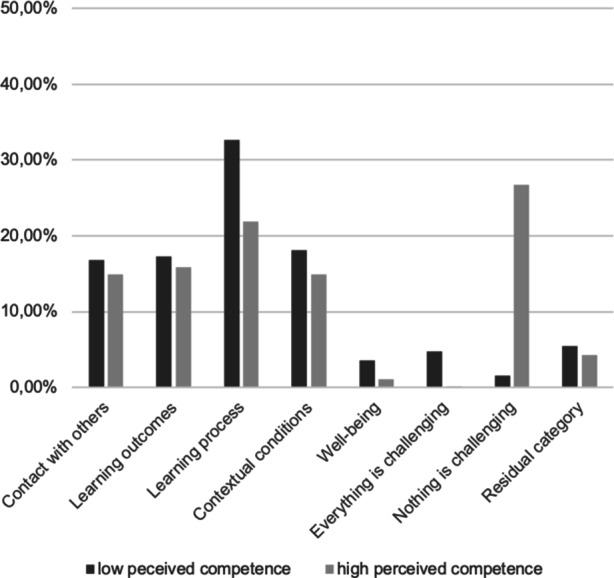
Overview over the differences in experienced challenges during distance learning between students who perceive themselves as high vs. low competent with respect to the main categories in the qualitative analysis

Overall, 23.54% of segments didn’t report any particular challenges in distance learning, with more such segments coded in the high perceived competence group compared to the low perceived competence group. On the other hand, 71.98% of coded segments concerned challenges students faced. Thereof, 0.74% were coded as *Everything feels challenging* right now, with more such answers coded in the low perceived competence group opposed to the high perceived competence group. Challenges concerning the *Learning process* were coded for 23.26% of answers (more often by the lacking competence group), whereas in 16.09% of the segments difficulties in achieving the desired *Learning outcomes *were mentioned. *Contextual conditions *and a lack of* Contact with others* were mentioned with approximately the same frequency (15.36% and 15.16%, respectively). In 1.38% of the coded segments, students reported that their physical and mental *Well-being* was challenged in the current situation. A further 4.48% of segments couldn’t be coded in any content-bearing category and were therefore assigned to the *Residual category*. Table I in the supplementary material provides a quantitative summary of coded segments (absolute and relative frequencies for main and subcategories) for all students as well as separately with high vs. low perceived competence. An overview over the differences between high vs. low perceived competence students regarding the main categories is provided in Fig. 1.

##### *Contact with others*

Within this category, lack of contact with or support from teachers was mentioned most frequently by students with both high and low perceived competence. Students reported that they missed face-to-face communication with teachers in real life as well as online, stating that they often didn’t understand the instructions and assignments and that they needed more explanations—particularly when learning new content. Moreover, students stated that they had difficulty contacting teachers and were getting no or delayed answers to their questions. In addition, both groups mentioned a lack of feedback from teachers. Consequently, they didn’t know whether their performance was sufficient. Some students also indicated that they would like to have more contact with their peers, either in order to support each other in school-related efforts or simply because they missed their friends. Whereas students lacking perceived competence often referred to feeling left alone with the material (“That you can’t really ask anybody”) and not getting help in general, only students in the high perceived competence group reported that their parents and siblings couldn’t support them, either because they were busy or because they lacked the skills to do so.

##### *Learning outcomes*

Students from both groups indicated difficulties in achieving learning outcomes, especially in terms of learning and understanding new topics. In this context, challenges in specific subjects were often mentioned. Above all, mathematics seemed to be particularly challenging. Some students stated that tasks in non-core subjects distracted them from completing their work for more important subjects like mathematics and that they wished that “music lessons, arts, cooking, handicraft lessons etc. would currently simply be omitted or reduced”.

##### *Learning process*

More segments related to the learning process were coded in the low perceived competence group compared to the high perceived competence group. Difficulties in organizing the learning process were reported most often. Whereas a lack of daily structure was only of concern to a few students, a greater percentage of segments addressed difficulties in keeping track of all tasks to be done, managing tasks and time and adhering to deadlines. Difficulties concentrating and avoiding distractions as well as a lack of motivation and (self-)discipline were mentioned more often in the low perceived competence group. While learning independently and without support was difficult for both groups, it was even more challenging for students who perceived themselves as lacking competence.

##### *Contextual conditions*

In terms of contextual conditions, challenging school-related requirements were mentioned most often by students, especially in the low perceived competence group. Students reported that they had too many assignments to do in too little time, with even more time pressure than during regular schooling (“Many teachers assign many more tasks than one would normally manage in school during this period”). Some students mentioned that their home learning environment was suboptimal, as they had to share a room with siblings or were otherwise distracted while studying. More segments related to the digital learning environment itself were coded in the high perceived competence group. Difficulties arose mainly with respect to the learning platforms, either because there were too many different platforms that had to be checked, running the risk of overlooking important information or assignments, or because the platforms didn’t work. In addition, some students reported technical issues due to malfunctioning soft- or hardware or because they lacked the necessary technical equipment for digital learning (e.g., they had to share a computer with another family member or had no access to a printer). Students also mentioned that they didn’t have sufficient knowledge to accomplish all necessary tasks (e.g., receiving and handing in digital assignments) and that they weren’t accustomed to working in front of a screen for prolonged periods of time and were easily distracted and exhausted, e.g., “Constantly looking at a screen (I have constant headaches from it despite taking breaks).”

##### *Well-being*

Challenges in maintaining one’s physical and mental well-being were coded more frequently in the group of students who perceived themselves as less competent, who mentioned dealing with general anxiety and uncertainty resulting from the situation and struggling to maintain a healthy learning-life balance. Students also indicated that being in close quarters with their family led to increased stress and arguments. The lack of opportunities to move around was mentioned by a few students.

#### Successes in distance learning

**Fig. 2:**
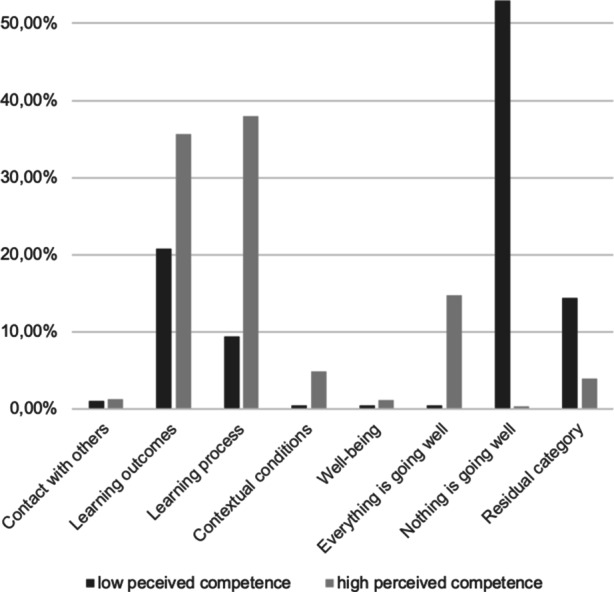
Overview over the difference in experiences success during distance learning between students who perceive themselves as high vs. low competent with respect to the main categories in the qualitative analysis

Overall, in 91.96% of coded segments, students reported that there was something positive to gain in distance learning, with 13.90% stating that *Everything is going well* right now. Notably, there was a large difference in coded segments between those students who perceived themselves as low (0.50%) compared to high competent (14.76%). While 52.97% of coded segments in the lacking perceived competence group were assigned the code *Nothing is going well*, only 0.32% of the coded segments in the highly perceived competent group were coded in this way (out of a total of coded segments 3.48%). In more detail, success in terms of the *Learning process *was the most frequently mentioned subcategory (36.29%), again with low perceived competence students mentioning this category less often (9.41%) than high perceived competence students (38.01%). The second most frequently coded category concerned *Success in Achieving learning outcomes* (34.80%). Segments in this category were coded less often in the low perceived competence group (20.79%) than in the high perceived competence group (35.67%). Good *Contextual conditions *were addressed in 4.64% of coded segments, with students who perceived themselves as lacking in perceived competence mentioning this form of success less often (0.50%) than students who perceived themselves as highly competent (4.91%). The remaining two categories, receiving support from and staying in *Contact with others* (1.25%) and successfully maintaining one’s *Well-being* (1.07%) were mentioned almost exclusively by students who perceived themselves as highly competent, with only 2 and 1 coded segment(s), respectively, in these categories found in the low perceived competence group. For this question, 4.55% of the segments couldn’t be coded in any content-bearing category and were assigned to the *Residual category*. A quantitative summary of all coded segments is provided in Table II in the supplementary material. Fig. 2 gives an overview over the differences between students who perceive themselves as highly vs. lacking competence with respect to the main categories.

##### *Contact with others*

Within this subcategory, staying in *Contact with teachers* and receiving teacher support were coded most frequently and only in the high perceived competence group. Students stated that they were able to contact teachers and ask questions, enjoyed their (online) lessons and received timely (and often positive) feedback on assignments. Some students also addressed that they successfully learned with peers (e.g., group work).

##### *Learning outcomes*

Students from both groups mentioned that learning in specific subjects or tasks was going well. Students who perceived themselves as highly competent reported more frequently that they were successfully completing tasks and assignments, noting that they were working more thoroughly and thus making fewer mistakes or else being faster and more productive. Students from the highly perceived competence also indicated that they were able to understand new material and got better grades in distance learning; in contrast, only two segments in the low perceived competence group fell within these subcategories.

##### *Learning process*

Concerning their learning process, students who perceived themselves as lacking competence described fewer successes in learning independently than students from the high perceived competence group. Although both groups mentioned that they were successful at setting priorities and learning at their own pace, students in the high perceived competence group indicated that they were more self-reliant in their learning overall and even enjoyed learning independently (“I actually like doing things on my own”). Students in the low perceived competence group indicated less often that they were successful in being organized, especially with respect to managing their time and planning their tasks as well as adhering to deadlines.

##### *Contextual conditions*

With regard to the learning context, only one segment was coded in the low perceived competence group, which addressed successfully working online (“that I can do many online exercises or that some things are well explained on YouTube”). Students from the high perceived competence group, in contrast, indicated that they enjoyed the quiet learning environment at home and that they felt successful in digital learning. They mentioned getting better at working on the computer (“Handling of the computer has improved”) and taking advantage of the benefits of technologies (“Writing texts on the computer, because it is much easier to change things”).

##### *Well-being*

Only one segment in the low perceived competence group was assigned to this category (“The balance between breaks and learning”). A few students from the high perceived competence group stated that they were successfully maintaining their well-being, mainly by taking breaks while learning and maintaining a healthy life-learning balance overall.

#### Need for support

**Fig. 3:**
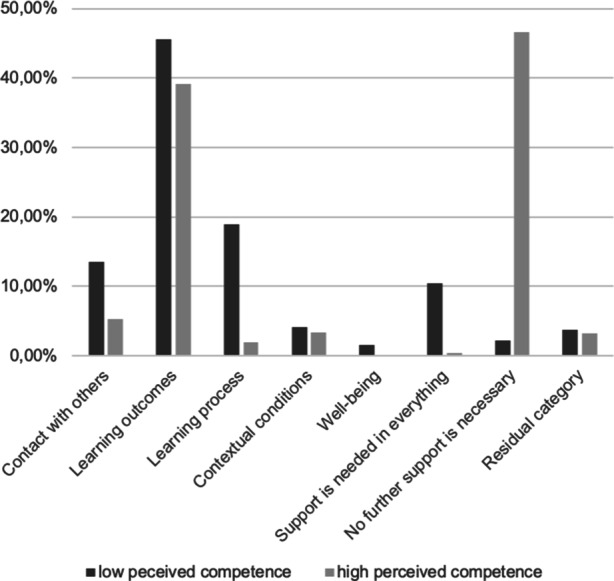
Overview over the differences in the need for support during distance learning between students who perceive themselves as high vs. low competent with respect to the main categories in the qualitative analysis

Overall, in 55.56% of coded segments, students indicated that they needed support in distance learning, of which 1.69% stated that *Support is needed in everything*. The number of coded segments in relation to group size differed between students who perceived themselves as high vs. lacking competence. In total, 40.94% of students indicated that they needed no further support, either because they already felt sufficiently supported or because they did not need any support at all. Notably, only 2.20% of segments in the low perceived competence group were coded thusly, compared to 46.61% in the high perceived competence group. The same gap can be observed in almost all the main categories, specifically for need for support with the *Learning process* (4.10%) as well as in the need for psychological *Well-being *(0.20%) and *Contact with others* (6.31%). Only with respect achieving* Learning outcomes* (40.02%) and in dealing with *Contextual conditions* did both groups expressed the same desire for further support (3.42%). Finally, 3.29% of the segments couldn’t be coded in any content-bearing category and were assigned to the *Residual category*. A quantitative summary of all segments can be found in Table III in the supplementary material; a narrative summary is provided below. In Fig. 3 the differences with respect to the main categories between students who perceive themselves as high vs. low competent is provided.

##### *Contact with others*

Very few segments referred to the desire for (more) contact with or support from family, parents or guardians and peers; a few segments mentioned the need for contact with or support from teachers. Several students noted that they would like to have regular face-to-face meetings (e.g., “my teachers whom I can address directly in class”), in order to get clear instructions. Students from the low perceived competence group particularly missed having a teacher who could explain things to them (e.g., “Perhaps that the topics can be explained in more detail than the book says”).

##### *Learning outcomes*

In most of the segments within this category, students expressed a desire for support in learning specific subjects, especially mathematics but also German and English, often with respect to learning new material (e.g., “It is difficult to understand the new material”).

##### *Learning process*

Regarding their learning process, students particularly expressed a need for support in staying organized, specifically in terms of keeping track of their tasks and in managing their tasks and time. They also needed support in maintaining motivation and self-discipline, stating, for example, that they would like to have a “motivational trainer” to help them get started and finish their tasks. This was mentioned more frequently in the low perceived competence group, which indicates a greater need for support among students with low perceived competence.

##### *Contextual conditions*

Both groups mentioned contextual conditions as an area where they would need support, especially in regard to the digital learning environment, where some students expressed that they would like to have help “when dealing with the computer” in general, with “[…] finding your way around the platforms” and with technical issues like their Internet connection (“Better and more stable Internet”). Students also indicated that they needed help with too demanding school requirements, hoping for “fewer tasks” and “more time” to accomplish them.

##### *Well-being*

Need for help with psychological well-being was expressed only by students in the low perceived competence group. A total of five segments were coded, all referring to the desire to have less stress in general, needing help in dealing with aggression and generally feeling lost and hopeless (“Through all the events, the goal disappears in front of your eyes and you sometimes ask yourself what the point is of going further, because at the moment everything seems hopeless”).

## Discussion

The present study aimed to gain insight into how students coped with the unique and challenging situation of distance learning during COVID-19 pandemic by investigating SRL, intrinsic motivation and passive procrastination. The large number of participants in our study allowed us to examine subsamples of students who perceived themselves as particularly high or low in competence. By complementing the quantitative data with open-ended questions, we were able to investigate differing underlying mechanisms in these two groups.

Our first research question addressed the differences in regard to self-regulated learning, intrinsic learning motivation and passive procrastination between students who perceived themselves as high vs low in competence. A quantitative approach was used to answer this question. As expected, our results showed that students with high perceived competence are better able to manage their time and tasks and plan their goals, use metacognitive strategies more often and have higher intrinsic motivation than students with low perceived competence. Our results are in line with previous studies finding perceived competence to be positively related to various aspects of SRL, such as planning and goal setting, time management and metacognitive strategies (Miller et al. 1993; Lüftenegger et al. 2012; Zimmerman and Martinez-Pons 1986). Moreover, as hypothesized, students who perceived themselves as less competent exhibited higher passive procrastination. This result is in accordance with findings of Haghbin et al. (2012) and Brando-Garrido et al. (2020), who found that perceived competence was negatively associated with procrastination in university students. However, we expand these previous findings by examining a younger age group in a distance learning setting.

In our second research question, we focused on challenges, successes and areas where support is needed among students in this new learning situation. Again, we were interested in differences between students who perceived themselves as high vs. low in competence with respect to the current distance learning situation and if and how different mechanisms came into play in these two groups. A qualitative approach was taken to answer this research question. The thematic analysis of the open-ended questions largely complemented the results from our first research question, however students also mentioned new aspects (e.g., regarding their well-being) that weren’t included in the quantitative analysis (e.g., regarding their well-being) and offer additional insights into students’ experiences during distance learning.

In general, the qualitative analysis revealed that even though all students faced similar challenges in distance learning, students who perceived themselves as highly competent were better able to cope with the situation. For instance, whereas more students from the low perceived competence group stated that everything is challenging right now, students who perceived themselves as highly competent mentioned that everything is going well more frequently. Whereas both groups indicated similar challenges regarding, for example, understanding specific tasks, subjects and new material, students in the high perceived competence group more often reported being successful with learning independently and even enjoying their increased self-reliance. They also actively utilized the unique characteristics of distance learning (e.g., doing tasks on the computer) and more frequently reported getting better grades than in traditional school settings, partly because they could learn at their own pace and in their own time. Both groups acknowledge challenges with respect to organizing their learning and had a particularly hard time keeping track of tasks, managing their time and adhering to deadlines. However, students from the high perceived competence group indicated that they were more successful in dealing with these challenges, whereas low perceived competence students required more support. These findings further support the importance of SRL strategies for learning success and strengthen the results of our quantitative analysis, particularly regarding planning and time management but also with respect to metacognitive strategies such as monitoring goal attainment. Additionally, students from the low perceived competence group reported less motivation and self-discipline, stating that they needed support in starting and following through with tasks. This highlights the interplay between motivational and self-regulatory mechanisms in passive procrastination (Klingsieck 2013; Steel 2007), which was also reflected in the moderate to high correlations between the SRL-related scales, motivation and passive procrastination.

Both groups described their lack of contact with others as challenging; they missed their peers and required more opportunities for synchronized online teaching. This is in line with theoretical approaches like self-determination theory (Deci and Ryan 2008), where social relatedness as one of three basic psychological needs is considered to be essential for students’ intrinsic motivation, learning engagement and overall well-being. Moreover, students also had a hard time understanding instructions, complained about having to wait for answers, and emphasized the importance of direct teacher support. However, even if challenging, students in the high perceived competence group more often reported being successful in maintaining contact with teachers and peers. This is in line with the results of a study by Zimmerman and Martinez-Pons (1986), in which high-achieving students tended to utilize social resources and assistance more readily than low-achieving students. Actively seeking support may be a strategy primarily applied by students with high perceived competence, even though such a strategy may only be successful if teachers and other adults are available to respond. Consistent with the literature on antecedents of online learning success, our findings emphasize the importance of social support and integration (particularly teacher-student relations) in distance learning settings (Borup et al. 2014; Weiner 2003). Furthermore, Lock et al. (2017) propose that purposeful planning is necessary to create a distance learning environment that fosters SRL. However, due to the rapid implementation of school closures, teachers and students had to adapt to the new situation within a very short amount of time. Our results indicate that students who perceive themselves as highly competent might be better able to develop the necessary SRL skills on their own, whereas students from the low perceived competence group needed more support in learning how to regulate their learning.

With respect to contextual challenges, both groups felt that there were too many assignments to complete in too little time and that the amount of work had increased, compared to regular school, especially in non-core subjects. While some students reported that learning at their own pace enabled them to work diligently and effectively, the wish for additional time and/or fewer assignments was expressed particularly often by the low perceived competence group. This again emphasized the importance of well-established communication and feedback systems, not only from teacher to student but vice versa (Borup et al. 2014; Weiner 2003), as well as coordination among teachers so that the students’ cumulative workload for students can be assessed accurately.

Finally, students from the low perceived competence group also had more difficulty maintaining their physical and psychological well-being and stated that they felt anxious in this highly uncertain situation. Although not the primary subject of our study and therefore not included in the quantitative analysis, this finding coincides with theories on the relations between perceived competence, autonomy and well-being (Niemiec and Ryan 2009).

In summary, our study substantially contributes to current research as it underlines the importance of perceived competence for successfully coping in the difficult, stressful situation created by emergency distance learning. It draws attention to the role of perceived competence for positive learning behaviors like the use of SRL strategies and the avoidance of passive procrastination. Finally, perceived competence also positively affects intrinsic motivation, which has been found to be particularly important in distance learning settings. Furthermore, the qualitative analysis emphasizes the importance of social support and contact with teachers as well as peers, not only for developing important SRL skills but also for student’s well-being.

### Limitations and strengths of our study

Like all research, our study has several limitations. Due to the contact restrictions during the COVID-19 pandemic, we had to rely on self-report measures via an online questionnaire. We are fully aware that this approach comes with several caveats. Firstly, self-reports are always contingent on participants answering candidly. However, since we assured full anonymity and our questions did not target particularly sensitive topics, we are confident that students responded honestly. Secondly, data collection via online questionnaires excludes some (high-risk) populations (e.g., those without internet access, those who are not sufficiently proficient in the German language to understand our instructions and questions and those who have learning disabilities). While this is a problem faced by many studies, it may be of greater concern in this particular context (learning during COVID-19). We must assume that our results are positively biased and that the differences between students who perceive themselves as high in vs. lacking competence are probably graver in reality than our study suggests. Thirdly, despite the wide reach of our questionnaire, we didn’t collect data systematically but relied on various channels to promote participation in our study (see methods section). Therefore, we did not control the selection of our sample regarding sociodemographic variables (e.g., our sample turned out to be predominantly female). For these reasons, our study is not representative, and our results cannot be generalized to the larger Austrian student population.

However, our study also has several valuable aspects. Firstly, the current situation (almost all students in distance learning) allowed us to collect a very large sample, which in turn enabled us to identify and work with data on actual extreme groups while still retaining a considerable sample size. Moreover, supplementing the quantitative results with qualitative analysis provided deeper insights into the challenges but also opportunities of distance learning in students’ own words and—most importantly—allowed us to collect concrete information about the areas in which students express a need for support.

### Implications for distance learning

Although the generalizability of our results is somewhat limited due to the cross-sectional nature of the study and the sampling method, our findings underscore the relevance of SRL in autonomous learning situations, such as distance learning. Fostering SRL should therefore be made a priority in the physical classroom as well as in online teaching. SRL can be supported in various ways, such as helping students set goals and schedule their time or else supporting their monitoring by asking prompting questions (e.g., Dignath and Büttner 2008; Dresel and Haugwitz 2008; Stebner et al. 2020; Zimmerman and Martinez-Pons 1990). Helping students to set and reach achievable goals, allows them to experience increased perceived competence, which in turn also boosts intrinsic motivation and learning success (Ryan and Deci 2000). Introducing accountability partners may serve to foster social relations between students, in addition to encouraging them to follow through on their plans. In our study, students from both the high and low perceived competence groups expressed a desire for clear and comprehensive instructions for tasks and assignments. Teachers should provide detailed instructions, offer explanations when necessary and be available for questions if possible. In addition, the multitude of communication platforms used for e‑learning assignments and the different delivery modalities used by teachers overwhelmed and confused many students. Thus, better coordination among teachers regarding platforms but also deadlines and delivery intervals may relieve some of the stress students reported. Additionally, providing timely and respectful feedback can support students’ self-efficacy and motivation as well as the teacher-student relationship (Wisniewski et al. 2020). Some students expressed a need for social contact, which was particularly impaired during the COVID-19 lockdown. The need for social relatedness is identified as a basic psychological need within social determination theory (Deci and Ryan 1993, 2000, 2008). Therefore, enhancing social interaction by providing opportunities for synchronous (e.g., video conferences, virtual learning groups) as well as asynchronous (e.g., group work and forum discussions) communication may promote distance learning success (Broadbent and Poon 2015) and benefit students’ overall well-being (Ryan and Deci 2000). However, online communication also has drawbacks and must be mediated by teachers to reach its positive potential (Rovai 2007). Finally, even though digital learning has been complementing traditional learning for years now (Brandhofer et al. 2019; Huber et al. 2020), most teachers were not prepared for the abrupt switch to distance learning that was necessary due to the COVID-19 regulations (Huber et al. 2020; World education blog 2020). Online teaching is not merely about transferring normal lessons into a digital environment; it requires unique skills that both teachers and students need to develop (Christensen and Alexander 2020; Flores 2020; Quiroz et al. 2016). Immediate action should be taken to better prepare and support teachers for distance and online education (Whalen 2020).

### Conclusion and future direction

Although distance learning and in particular emergency distance learning in a crisis such as COVID-19 present challenges for students as well as teachers and parents, SRL skills and high intrinsic motivation may serve as protective factors and foster not only learning success but also student’s well-being. Our findings are of particular interest in light of possible school closures in future crises but can also serve to inform stakeholders seeking to develop effective concepts for successful future blended or online learning.

Several areas of interest could be addressed in future research. Other important actors (e.g., parents, teachers, school principals) should be approached to gain an understanding of the distance learning situation from different perspectives. To gain additional knowledge about the differentiating effects of subjects and the way lessons and assignments are presented, further studies taking methods of delivery should be conducted. Additionally, a longitudinal design would allow for insights into changes in student’s perceived competence, self-regulated learning and motivation, providing further information about the underlying mechanisms that influence distance learning success. Furthermore, our study focused on student’s subjectively perceived competence as opposed to objective performance data. Future research could aim to incorporate other measures of academic success (e.g., grades or achievement tests). Finally, we assessed contact with others as a form of resource management in our metacognition scale. However, the results of the qualitative analysis suggest that contact with and support from teachers may play an essential role for SRL and intrinsic motivation and well-being, especially in distance learning. The role of social integration and support should therefore be investigated in future studies on distance learning.

## Supplementary Information

Table I. *Quantitative summary of Question 1: “What do you currently find especially hard when studying?”*

Table II. *Quantitative summary of Question 2: “What parts of studying are currently going particularly well?”*

Table III. *Quantitative summary of Question 3: “With what could you currently use some help?”*

Table IV. *Category system for Question 1: “What do you currently find especially hard when studying?”*

Table V. *Category system for Question 2: “What parts of studying are currently going particularly well?”*

Table VI. *Category system for Question 3: “With what could you currently use some help?”*

## References

[CR1] Adam NL, Alzahri FB, Cik Soh S, Bakar AN, Kamal MNA, Badioze Zaman H, Robinson P, Smeaton AF, Shih TK, Velastin S, Terutoshi T, Jaafar A, Mohamad NA (2017). Self-regulated learning and online learning: A systematic review. Advances in Visual Informatics.

[CR2] Borup J, Graham CR, Drysdale JS (2014). The nature of teacher engagement at an online high school. British Journal of Educational Technology.

[CR3] Bozkurt A, Jung I, Xiao J, Vladimirschi V, Schuwer R, Egorov G, Rodes V (2020). A global outlook to the interruption of education due to COVID-19 Pandemic: Navigating in a time of uncertainty and crisis. Asian Journal of Distance Education.

[CR4] Brandhofer G, Baumgartner P, Ebner M, Köberer N, Trültzsch-Wijnen C, Wiesner C (2019). Bildung im Zeitalter der Digitalisierung.

[CR5] Brando-Garrido C, Montes-Hidalgo J, Limonero JT, Gómez-Romero MJ, Tomás-Sábado J (2020). Relationship of academic procrastination with perceived competence, coping, self-esteem and self-efficacy in nursing students. *Enfermería Clínica (English*. Edition.

[CR6] Braun V, Clarke V (2006). Using thematic analysis in psychology. Qualitative research in psychology.

[CR7] Broadbent J, Poon WL (2015). Self-regulated learning strategies & academic achievement in online higher education learning environments: a systematic review. The Internet and Higher Education.

[CR70] Van den Broeck A, Vansteenkiste M, Witte H, Soenens B, Lens W (2010). Capturing autonomy, competence, and relatedness at work: construction and initial validation of the Work-related Basic Need Satisfaction scale. Journal of Occupational and Organizational Psychology.

[CR8] Cavanaugh C, Gillan KJ, Kromrey J, Hess M, Blomeyer R (2004). The effects of distance education on K-12 student outcomes: a meta-analysis.

[CR9] Chen KC, Jang SJ (2010). Motivation in online learning: testing a model of self-determination theory. Computers in Human Behavior.

[CR10] Cho Y, Weinstein CE, Wicker F (2011). Perceived competence and autonomy as moderators of the effects of achievement goal orientations. Educational Psychology.

[CR12] Christensen R, Alexander C (2020). Preparing K-12 Schools for a pandemic before it occurs. Journal of Technology and Teacher Education.

[CR11] Cohen J (1988). Statistical power analysis for the behavioral sciences.

[CR13] Dabbagh N, Kitsantas A (2004). Supporting self-regulation in student-centered web-based learning environments. International Journal on E-learning.

[CR17] Deci EL, Ryan RM (1985). Intrinsic motivation and self-determination in human behavior.

[CR16] Deci EL, Ryan RM (1993). Die Selbstbestimmungstheorie der Motivation und ihre Bedeutung für die Pädagogik. Zeitschrift für Pädagogik.

[CR15] Deci EL, Ryan RM (2000). The “what“” and “why” of goal pursuits: human needs and the self-determination of behavior. Psychological inquiry.

[CR14] Deci EL, Ryan RM (2008). Self-determination theory. Canadian Psychology = Psychologie Canadienne.

[CR18] Dent AL, Koenka AC (2016). The relation between self-regulated learning and academic achievement across childhood and adolescence: a meta-analysis. Educational Psychology Review.

[CR19] Dignath C, Büttner G (2008). Components of fostering self-regulated learning among students. A meta-analysis on intervention studies at primary and secondary school level. Metacognition Learning.

[CR20] Donker AS, De Boer H, Kostons D, Van Ewijk CD, van der Werf MP (2014). Effectiveness of learning strategy instruction on academic performance: a meta-analysis. Educational Research Review.

[CR21] Dresel M, Haugwitz M (2008). A computer-based approach to fostering motivation and self-regulated learning. The Journal of Experimental Education.

[CR22] European Commission, Directorate-General for Education, Youth, Sport and Culture (2018). Study on supporting school innovation across Europe. Final report.

[CR23] Federal Ministry of Education (2020a). BMBWF: Mehr als sechsmal so viele Kinder in schulischer Betreuung als vor Ostern. https://www.bmbwf.gv.at/Ministerium/Presse/20200423.html.. Accessed: 7. June 2020.

[CR24] Federal Ministry of Education (2020b). Coronavirus (COVID-19).https://www.bmbwf.gv.at/Ministerium/Informationspflicht/corona.html. Accessed: 7. June 2020.

[CR25] Ferla J, Valcke M, Schuyten G (2010). Judgments of self-perceived academic competence and their differential impact on students’ achievement motivation, learning approach, and academic performance. European Journal of Psychology of Education.

[CR26] Flores MA (2020). Preparing teachers to teach in complex settings: opportunities for professional learning and development. European Journal of Teacher Education.

[CR27] Fortier MS, Vallerand RJ, Guay F (1995). Academic motivation and school performance: toward a structural model. Contemporary Educational Psychology.

[CR28] Froiland JM, Oros E (2014). Intrinsic motivation, perceived competence and classroom engagement as longitudinal predictors of adolescent reading achievement. Educational Psychology.

[CR29] Fryer LK, Bovee HN (2016). Supporting students’ motivation for e-learning: teachers matter on and offline. The Internet and Higher Education.

[CR30] Fryer LK, Bovee HN, Nakao K (2014). E-learning: reasons students in language learning courses don’t want to. Computers & Education.

[CR31] Gillet N, Vallerand RJ, Lafrenière MAK (2012). Intrinsic and extrinsic school motivation as a function of age: the mediating role of autonomy support. Social Psychology of Education.

[CR33] Glöckner-Rist A, Engberding M, Höcker A, Rist F (2014). Prokrastinationsfragebogen für Studierende (PFS). Zusammenstellung sozialwissenschaftlicher Items und Skalen (ZIS).

[CR32] Gottfried AE, Fleming JS, Gottfried AW (2001). Continuity of academic intrinsic motivation from childhood through late adolescence: a longitudinal study. Journal of Educational Psychology.

[CR34] Guay F, Boggiano AK, Vallerand RJ (2001). Autonomy support, intrinsic motivation, and perceived competence: conceptual and empirical linkages. Personality and Social Psychology Bulletin.

[CR35] Haghbin M, McCaffrey A, Pychyl TA (2012). The complexity of the relation between fear of failure and procrastination. Journal of Rational-Emotive & Cognitive-Behavior Therapy.

[CR36] Howell AJ, Watson DC (2007). Procrastination: associations with achievement goal orientation and learning strategies. Personality and Individual Differences.

[CR37] Hu L, Bentler PM (1999). Cutoff criteria for fit indexes in covariance structure analysis:  conventional criteria versus new alternatives. Structural Equation Modeling: A Multidisciplinary Journal.

[CR39] Huber SG, Helm C (2020). Educational Assessment, Evaluation and Accountability.

[CR38] Huber SG, Günther PS, Schneider N, Helm C, Schwander M, Schneider J, Pruitt J (2020). COVID-19 und aktuelle Herausforderungen in Schule und Bildung.

[CR40] IBM (2017). IBM SPSS Statistics for Windows (25.0).

[CR42] Klingsieck KB (2013). Procrastination: When good things don’t come to those who wait. European Psychologist.

[CR41] Klingsieck KB (2018). Kurz und knapp – die Kurzskala des Fragebogens „Lernstrategien im Studium“ (LIST). Zeitschrift Für Pädagogische Psychologie.

[CR43] Lee M, Figueroa R (2012). Internal and external indicators of virtual learning success: a guide to success in K-12 virtual learning. Distance Learning.

[CR44] Lock J, Eaton SE, Kessy E (2017). Fostering self-regulation in online learning in K-12 education. Northwest Journal of Teacher Education.

[CR45] Lüftenegger M, Schober B, Van de Schoot R, Wagner P, Finsterwald M, Spiel C (2012). Lifelong learning as a goal—do autonomy and self-regulation in school result in well prepared pupils?. Learning and Instruction.

[CR46] Means B, Toyama Y, Murphy R, Baki M (2013). The effectiveness of online and blended learning: a meta-analysis of the empirical literature. Teachers College Record.

[CR47] Miller RB, Behrens JT, Greene BA, Newman DE (1993). Goals and perceived ability: Impact on student valuing, self-regulation, and persistence. Contemporary educational psychology.

[CR48] Mupinga DM (2005). Distance education in high schools: benefits, challenges, and suggestions. The Clearing House: A Journal of Educational Strategies, Issues and Ideas.

[CR49] Muthén LK, Muthén BO (2017). Mplus user’s guide.

[CR51] Niemiec CP, Ryan RM (2009). Autonomy, competence, and relatedness in the classroom: applying self-determination theory to educational practice. Theory and research in Education.

[CR50] Ning HK, Downing K (2010). The reciprocal relationship between motivation and self-regulation: a longitudinal study on academic performance. Learning and Individual Differences.

[CR52] Paechter M, Maier B (2010). Online or face-to-face? Students’ experiences and preferences in e-learning. The Internet and Higher Education.

[CR88] Panadero E (2017). A review of self-regulated learning: Six models and four directions for research. Frontiers in Psychology.

[CR53] Pichardo C, Justicia F, de la Fuente J, Martínez-Vicente JM, Berbén ABG (2014). Factor structure of the Self-Regulation Questionnaire (SRQ) at Spanish universities. The Spanish Journal of Psychology.

[CR54] Quiroz RE, Ritter NL, Li Y, Newton RC, Palkar T (2016). Standards based design: teaching K-12 educators to build quality online courses. Journal of Online Learning Research.

[CR55] Rakes GC, Dunn KE (2010). The impact of online graduate students’ motivation and self-regulation on academic procrastination. Journal of Interactive Online Learning.

[CR56] Raykov T (2009). Evaluation of scale reliability for unidimensional measures using latent variable modeling. Measurement and Evaluation in Counseling and Development.

[CR57] Reeve J, Nix G, Hamm D (2003). Testing models of the experience of self-determination in intrinsic motivation and the conundrum of choice. Journal of Educational Psychology.

[CR58] Rice KL (2006). A comprehensive look at distance education in the K-12 context. Journal of Research on Technology in Education.

[CR59] Rovai AP (2007). Facilitating online discussions effectively. The Internet and Higher Education.

[CR60] Ryan RM, Deci EL (2000). Intrinsic and extrinsic motivation: classic definitions and new directions. Contemporary Educational Psychology.

[CR62] Schober B, Lüftenegger M, Spiel C (2020). Learning conditions during COVID-19 Pupils (SUF edition).

[CR61] Schrenk R (2020). Distance Learning mit Moodle – Aktuelles aus Österreichs Schulen. GW Unterricht.

[CR63] Simonson, M., & Berg, G. (2016). Distance learning. Retrieved June 1, 2020, from https://www.britannica.com/topic/distance-learning. Accessed: 1. June 2020.

[CR64] Stebner F, Schuster C, Weber X-L, Roelle J, Wirth J, van Vorst H, Sumfleth E (2020). Indirekte Förderung des selbstregulierten Lernens – Praxistipps für den Fachunterricht. Von Sprosse zu Sprosse. Innovative Erarbeitung des Bohr’schen Atomkonzepts mit der Lernleiter.

[CR65] Steel P (2007). The nature of procrastination: a meta-analytic and theoretical review of quintessential self-regulatory failure. Psychological Bulletin.

[CR66] Steel P, Klingsieck K (2016). Academic procrastination: psychological antecedents revisited. Australian Psychologist.

[CR67] Stephan Y, Caudroit J, Boiché J, Sarrazin P (2011). Predictors of situational disengagement in the academic setting: the contribution of grades, perceived competence, and academic motivation. British Journal of Educational Psychology.

[CR68] Thomas AE, Müller FH, Bieg S (2018). Entwicklung und Validierung der Skalen zur motivationalen Regulation beim Lernen im Studium (SMR-LS). Diagnostica.

[CR69] UNICEF (2020). UNICEF scales up support in 145 countries to keep children learning, as COVID-19 forces majority of schools worldwide to close. https://www.unicef.org/press-releases/unicef-scales-support-145-countries-keep-children-learning-covid-19-forces-majority. Accessed: 15. May 2020.

[CR71] VERBI Software (2019). MAXQDA 2020 [computer software].

[CR72] Viner RM, Russell SJ, Croker H, Packer J, Ward J, Stansfield C, Mytton CB, Booy R (2020). School closure and management practices during coronavirus outbreaks including COVID-19: a rapid systematic review. The Lancet Child & Adolescent Health.

[CR74] Wahlmüller-Schiller C (2017). Bildung 4.0 – der Weg in die Zukunft. E & I Elektrotechnik und Informationstechnik.

[CR75] Wang C, Hsu HCK, Bonem EM, Moss JD, Yu S, Nelson DB, Levesque-Bristol C (2019). Need satisfaction and need dissatisfaction: a comparative study of online and face-to-face learning contexts. Computers in Human Behavior.

[CR76] Wang C-H, Shannon DM, Ross ME (2013). Students’ characteristics, self-regulated learning, technology self-efficacy, and course outcomes in online learning. Distance Education.

[CR77] Weiner C (2003). Key ingredients to online learning: adolescent students study in cyberspace—the nature of the study. International Journal on Learning.

[CR73] Whalen J (2020). Should teachers be trained in emergency remote teaching? Lessons learned from the COVID-19 pandemic. Journal of Technology and Teacher Education.

[CR78] WHO (2020). Timeline of WHO’s response to COVID-19. Retrieved July 3, 2020, from https://www.who.int/news-room/detail/29-06-2020-covidtimeline. Accessed: 3. July 2020.

[CR79] Wisniewski B, Zierer K, Hattie J (2020). The power of feedback revisited: a meta-analysis of educational feedback research. Frontiers in Psychology.

[CR80] Wolters CA (2003). Regulation of motivation: evaluating an underemphasized aspect of self-regulated learning. Educational psychologist.

[CR81] World Education Blog (2020, April 16). Charlotte, a teacher from Marseille: “We’re being asked to do a completely different job from before.” [Blog post]. https://www.spring.org.uk/the1sttransport. Accessed: 10. June 2020.

[CR82] Zeidner M, Boekaerts M, Pintrich PR, Boekaerts M, Pintrich PR, Zeidner M (2000). Self-regulation: Directions and challenges for future research. Handbook of self-regulation.

[CR87] Zimmerman BJ, Boekaerts M, Pintrich PR, Zeidner M (2000). Attaining self-regulation: A social cognitive perspective. Handbook of Self-Regulation.

[CR83] Zimmerman BJ (1990). Self-regulated learning and academic achievement: an overview. Educational Psychologist.

[CR84] Zimmerman BJ, Martinez-Pons M (1990). Student differences in self-regulated learning: relating grade, sex, and giftedness to self-efficacy and strategy use. Journal of Educational Psychology.

[CR85] Zimmerman BJ, Pons MM (1986). Development of a structured interview for assessing student use of self-regulated learning strategies. American Educational Research Journal.

[CR86] Zisimopoulos DA, Galanaki EP (2009). Academic intrinsic motivation and perceived academic competence in Greek elementary students with and without learning disabilities. Learning Disabilities Research & Practice.

