# Supplementary material for: Learning during COVID-19: the role of self-regulated learning, motivation, and procrastination for perceived competence
Source: Z Erziehwiss. 2021 Mar 4;24(2):393–418. doi: 10.1007/s11618-021-01002-x (PMC7931168; doi:10.1007/s11618-021-01002-x)
Supplement: Supplementary file 1 — Table I. Quantitative summary of Question 1: “What do you currently find especially hard when studying?” [file 11618_2021_1002_MOESM1_ESM.docx]

| Table I  *Quantitative summary of Question 1: “What do you currently find especially hard when studying?”* | | | | | | | | |
| --- | --- | --- | --- | --- | --- | --- | --- | --- |
|  | **low competence** | | **high competence** | | **total** | | ***χ²*** | ***p*** |
|  | sum | rel. % | sum | rel. % | sum | rel. % |  |  |
| **1. Contact with others** | **67** | **16.79%** | **407** | **14.92%** | **474** | **15.16%** | **0.943** | **.331** |
| *1.1. Lack of contact/support with/from others in general* | *12* | *3.01%* | *5* | *0.18%* | *17* | *0.54%* | *51.328* | *< .001** |
| *1.2. Lack of contact/support with/from guardians/family* | *0* | *0.00%* | *14* | *0.51%* | *14* | *0.45%* | *2.058* | *.240** |
| *1.3. Lack of contact/support with/from peers* | *5* | *1.25%* | *32* | *1.17%* | *37* | *1.18%* | *0.019* | *.806** |
| *1.4. Lack of contact/support with/from teachers* | *50* | *12.53%* | *356* | *13.05%* | *406* | *12.99%* | *0.084* | *.771* |
| 1.4.1. Lack of contact/support with/from teachers in general | 14 | 3.51% | 65 | 2.38% | 79 | 2.53% | - | - |
| 1.4.2. Unclear/incomprehensible tasks | 9 | 2.26% | 65 | 2.38% | 74 | 2.37% | - | - |
| 1.4.3. Too few explanations | 16 | 4.01% | 87 | 3.19% | 103 | 3.29% | - | - |
| 1.4.4. Difficulties getting answers to questions | 8 | 2.01% | 114 | 4.18% | 122 | 3.90% | - | - |
| 1.4.5. Too little/late feedback | 2 | 0.50% | 25 | 0.92% | 27 | 0.86% | - | - |
| 1.4.6. Lack of understanding/empathy for the situation | 1 | 0.25% | 0 | 0.00% | 1 | 0.03% | - | - |
| **2. Learning outcomes** | **69** | **17.29%** | **434** | **15.91%** | **503** | **16.09%** | **0.490** | **.484** |
| *2.1. Challenges in completing assignments* | *1* | *0.25%* | *14* | *0.51%* | *15* | *0.48%* | *0.503* | *.709** |
| 2.1.1. Challenges in completing assignments in general | 0 | 0.00% | 3 | 0.11% | 3 | 0.10% | - | - |
| 2.1.2. Challenges in completing assignments diligently/accurately | 1 | 0.25% | 9 | 0.33% | 10 | 0.32% | - | - |
| 2.1.3. Challenges in completing assignments effectively/productively | 0 | 0.00% | 2 | 0.07% | 2 | 0.06% | - | - |
| *2.2. Challenges when learning and/or understanding (new) material* | *36* | *9.02%* | *194* | *7.11%* | *230* | *7.36%* | *1.860* | *.173* |
| *2.3. Challenges regarding preparation for the final exams (Matura)* | *2* | *0.50%* | *9* | *0.33%* | *11* | *0.35%* | *0.291* | *.641** |
| *2.4. Challenges regarding specific subjects/tasks/assignments* | *30* | *7.52%* | *217* | *7.96%* | *247* | *7.90%* | *0.092* | *.762* |
| 2.4.1. Challenges regarding specific subjects/tasks/assignment in general | 6 | 1.50% | 26 | 0.95% | 32 | 1.02% | - | - |
| 2.4.2. Challenges in working for (perceived) boring/unimportant subjects | 4 | 1.00% | 11 | 0.40% | 15 | 0.48% | - | - |
| 2.4.3. Challenges in Mathematics | 14 | 3.51% | 95 | 3.48% | 109 | 3.49% | - | - |
| 2.4.4. Challenges in German | 2 | 0.50% | 17 | 0.62% | 19 | 0.61% | - | - |
| 2.4.5. Challenges in English | 0 | 0.00% | 30 | 1.10% | 30 | 0.96% | - | - |
| 2.4.6. Challenges in other subjects | 4 | 1.00% | 38 | 1.39% | 42 | 1.34% | - | - |
| **3. Learning process** | **130** | **32.58%** | **597** | **21.89%** | **727** | **23.26%** | **22.283** | **<.001** |
| *3.1. Challenges regarding the learning process in general* | 0 | 0.00% | 2 | 0.07% | 2 | 0.06% | 0.293 | .761* |
| *3.2. Challenges when learning alone/independently* | 28 | 7.02% | 112 | 4.11% | 140 | 4.48% | 6.892 | .009 |
| *3.3. Difficulties concentrating/avoiding distractions* | 31 | 7.77% | 118 | 4.33% | 149 | 4.77% | 9.086 | .003 |
| *3.4. Motivational and volitional challenges* | 30 | 7.52% | 102 | 3.74% | 132 | 4.22% | 12.287 | < .001 |
| 3.4.1. Lack of motivation | 19 | 4.76% | 62 | 2.27% | 81 | 2.59% | - | - |
| 3.4.2. Lack of joy in learning | 0 | 0.00% | 1 | 0.04% | 1 | 0.03% | - | - |
| 3.4.3. Lack of (self-)discipline | 11 | 2.76% | 39 | 1.43% | 50 | 1.60% | - | - |
| *3.4. Difficulties in (self-)organization* | 41 | 10.28% | 263 | 9.64% | 304 | 9.72% | 0.158 | .691 |
| 3.4.1. Difficulties in (self-)organisation in general | 3 | 0.75% | 18 | 0.66% | 21 | 0.67% | - | - |
| 3.4.2. Difficulties due to the lack of daily structure | 1 | 0.25% | 12 | 0.44% | 13 | 0.42% | - | - |
| 3.4.3. Difficulties in managing tasks and time | 13 | 3.26% | 68 | 2.49% | 81 | 2.59% | 0.806 | .369 |
| 3.4.4. Difficulties keeping track of tasks to be done | 19 | 4.76% | 113 | 4.14% | 132 | 4.22% | - | - |
| 3.4.5. Difficulties adhering to deadlines | 5 | 1.25% | 52 | 1.91% | 57 | 1.82% | - | - |
| **4. Contextual conditions** | **72** | **18.05%** | **408** | **14.96%** | **480** | **15.36%** | **2.546** | **.111** |
| *4.1. Challenges due to poor learning materials* | 0 | 0.00% | 3 | 0.11% | 3 | 0.10% | 0.439 | > .999* |
| *4.2. Challenging learning environment* | 12 | 3.01% | 46 | 1.69% | 58 | 1.86% | 3.334 | .068 |
| *4.3. Challenging school requirements* | 41 | 10.28% | 148 | 5.43% | 189 | 6.05% | 14.404 | < .001 |
| 4.3.1. Too high school-related requirements | 38 | 9.52% | 145 | 5.32% | 183 | 5.85% | - | - |
| 4.3.1.1. ... due to too little coordination between the teachers | 2 | 0.50% | 8 | 0.29% | 10 | 0.32% | - | - |
| 4.3.1.2. ... due to too much time pressure | 9 | 2.26% | 15 | 0.55% | 24 | 0.77% | - | - |
| 4.3.1.3. ... due to too many/too demanding tasks | 27 | 6.77% | 122 | 4.47% | 149 | 4.77% | - | - |
| 4.3.2. Too low school-related requirements | 3 | 0.75% | 3 | 0.11% | 6 | 0.19% | - | - |
| *4.4. Challenges due to the digital learning setting* | 18 | 4.51% | 210 | 7.70% | 228 | 7.29% | 5.237 | .022 |
| 4.4.1. Teachers are not familiar with the necessary technology | 1 | 0.25% | 3 | 0.11% | 4 | 0.13% | - | - |
| 4.4.2. Receiving and handing in/doing (online) assignments | 2 | 0.50% | 23 | 0.84% | 25 | 0.80% | - | - |
| 4.4.3. Challenges due to digitally mediated teaching & learning | 4 | 1.00% | 21 | 0.77% | 25 | 0.80% | - | - |
| 4.4.4. Challenges working with communication platforms | 6 | 1.50% | 67 | 2.46% | 73 | 2.34% | - | - |
| 4.4.4.1. Too many different communication platforms | 6 | 1.50% | 37 | 1.36% | 43 | 1.38% | - | - |
| 4.4.4.2. Communication platforms don’t work | 0 | 0.00% | 30 | 1.10% | 30 | 0.96% | - | - |
| 4.4.5. Challenges when working on the computer | 0 | 0.00% | 20 | 0.73% | 20 | 0.64% | - | - |
| 4.4.6. Spending a lot of time in front of the computer | 3 | 0.75% | 26 | 0.95% | 29 | 0.93% | - | - |
| 4.4.7. Difficulties with the necessary equipment | 2 | 0.50% | 50 | 1.83% | 52 | 1.66% | - | - |
| 4.4.7.1. Technical equipment doesn’t work | 0 | 0.00% | 8 | 0.29% | 8 | 0.26% | - | - |
| 4.4.7.2. Not having the necessary equipment | 0 | 0.00% | 12 | 0.44% | 12 | 0.38% | - | - |
| 4.4.7.3. Problems with the Internet connection or (wireless) network | 2 | 0.50% | 30 | 1.10% | 32 | 1.02% | - | - |
| *4.5. Additional burdens due to other obligations or chores* | 1 | 0.25% | 1 | 0.04% | 2 | 0.06% | 2.492 | .239* |
| **5. Well-being** | **14** | **3.51%** | **29** | **1.06%** | **43** | **1.38%** | **15.342** | **< .001** |
| *5.1. Psychological challenges* | 12 | 3.01% | 25 | 0.92% | 37 | 1.18% | 13.009 | .001* |
| 5.1.1. Helplessness/anxiety/stress | 9 | 2.26% | 5 | 0.18% | 14 | 0.45% | - | - |
| 5.1.2. Dealing with uncertainty | 0 | 0.00% | 3 | 0.11% | 3 | 0.10% | - | - |
| 5.1.3. Learning-life balance | 1 | 0.25% | 14 | 0.51% | 15 | 0.48% | - | - |
| 5.1.4. Family difficulties | 2 | 0.50% | 3 | 0.11% | 5 | 0.16% | - | - |
| *5.2. Physical challenges* | 2 | 0.50% | 4 | 0.15% | 6 | 0.19% | 2.284 | .172 |
| **6. Everything is challenging** | **19** | **4.76%** | **4** | **0.15%** | **23** | **0.74%** | **101.513** | **< .001*** |
| **7. Nothing is challenging** | **6** | **1.50%** | **730** | **26.77%** | **736** | **23.54%** | **123.433** | **< .001** |
| **8. Residual Category** | **22** | **5.51%** | **118** | **4.33%** | **140** | **4.48%** | **1.146** | **.284** |
| Sum | 399 | 100.00% | 2727 | 100.00% | 3126 | 100.00% |  |  |
| N = Documents | 235 |  | 2417 |  | 2652 |  |  |  |
| *Note.* Percentages for the separate groups were calculated by relating the number of coded segments in a specific category to the number of coded segments in the group overall. Total percentages were related to the overall number of coded segments.  *In case of more than 20% of the cells having and expected absolute value < 5 Fisher’s Exact Test (2-sided) has been reported. | | | | | | | | |
