# Supplementary material for: Learning during COVID-19: the role of self-regulated learning, motivation, and procrastination for perceived competence
Source: Z Erziehwiss. 2021 Mar 4;24(2):393–418. doi: 10.1007/s11618-021-01002-x (PMC7931168; doi:10.1007/s11618-021-01002-x)
Supplement: Supplementary file 2 — Table II. Quantitative summary of Question 2: “What parts of studying are currently going particularly well?” [file 11618_2021_1002_MOESM2_ESM.docx]

| Table II  *Quantitative summary of Question 2: “What parts of studying are currently going particularly well?”* | | | | | | | | |
| --- | --- | --- | --- | --- | --- | --- | --- | --- |
|  | **low competence** | | **high competence** | | **total** | | ***χ²*** | ***p*** |
|  | sum | rel. % | sum | rel. % | sum | rel. % |  |  |
| **1. Contact with others** | **2** | **0.99%** | **40** | **1.27%** | **42** | **1.25%** | **0.118** | **> .999*** |
| *1.1. Receiving support from/keeping in contact with others in general* | *0* | *0.00%* | *2* | *0.06%* | *2* | *0.06%* | *0.128* | *> .999** |
| *1.2. Receiving support from/keeping in contact with guardians/family* | *0* | *0.00%* | *4* | *0.13%* | *4* | *0.12%* | *0.256* | *> .999** |
| *1.3. Receiving support from/keeping in contact with peers* | *2* | *0.99%* | *11* | *0.35%* | *13* | *0.39%* | *2.028* | *.182** |
| *1.4. Receiving support from/keeping in contact with teachers* | *0* | *0.00%* | *23* | *0.73%* | *23* | *0.68%* | *1.482* | *.396** |
| 1.4.1. Receiving support from/keeping in contact with teachers in general | 0 | 0.00% | 12 | 0.38% | 12 | 0.36% | - | - |
| 1.4.2. Teachers are giving clear/comprehensible instructions | 0 | 0.00% | 1 | 0.03% | 1 | 0.03% | - | - |
| 1.4.3. Teachers are giving comprehensive explanations | 0 | 0.00% | 3 | 0.10% | 3 | 0.09% | - | - |
| 1.4.4. Teachers are answering questions (in time) | 0 | 0.00% | 4 | 0.13% | 4 | 0.12% | - | - |
| 1.4.5. Teachers are giving (timely) feedback | 0 | 0.00% | 3 | 0.10% | 3 | 0.09% | - | - |
| **2. Learning outcomes** | **42** | **20.79%** | **1127** | **35.70%** | **1169** | **34.80%** | **26.384** | **< .001** |
| *2.1. Success in achieving learning outcomes in general* | *0* | *0.00%* | *13* | *0.41%* | *13* | *0.39%* | *0.835* | *> .999** |
| *2.2. Success in understanding assignments* | *0* | *0.00%* | *13* | *0.41%* | *13* | *0.39%* | *0.835* | *> .999** |
| *2.3. Success in completing assignments* | *7* | *3.47%* | *362* | *11.47%* | *369* | *10.99%* | *12.429* | *< .001* |
| 2.3.1. Success in completing assignments in general | 3 | 1.49% | 108 | 3.42% | 111 | 3.30% | - | - |
| 2.3.2. ... diligently/accurately | 1 | 0.50% | 108 | 3.42% | 109 | 3.25% | 4.943 | .026 |
| 2.3.3. ... effectively/productively | 3 | 1.49% | 146 | 4.62% | 149 | 4.44% | 4.414 | .036 |
| *2.4. Successfully learning (new) material* | *2* | *0.99%* | *193* | *6.11%* | *195* | *5.81%* | *9.113* | *.003* |
| *2.5. Successfully preparing for the final exams (“Matura”)* | *0* | *0.00%* | *2* | *0.06%* | *2* | *0.06%* | *0.128* | *> .999** |
| *2.6. Achieving learning outcomes in specific subjects/tasks/assignments* | *33* | *16.34%* | *519* | *16.44%* | *552* | *16.43%* | *0.001* | *.969* |
| 2.6.1. Success with specific subjects/tasks/assignments in general | 13 | 6.44% | 100 | 3.17% | 113 | 3.36% | - | - |
| 2.6.2. Achieving learning outcomes in Mathematics | 3 | 1.49% | 147 | 4.66% | 150 | 4.47% | - | - |
| 2.6.3. Achieving learning outcomes in German | 4 | 1.98% | 102 | 3.23% | 106 | 3.16% | - | - |
| 2.6.4. Achieving learning outcomes in English | 7 | 3.47% | 116 | 3.67% | 123 | 3.66% | - | - |
| 2.6.5. Achieving learning outcomes in other subjects | 6 | 2.97% | 54 | 1.71% | 60 | 1.79% | - | - |
| *2.7. Getting good/better feedback/grades* | *0* | *0.00%* | *25* | *0.79%* | *25* | *0.74%* | *1.612* | *.398** |
| **3. Learning process** | **19** | **9.41%** | **1200** | **38.01%** | **1219** | **36.29%** | **69.589** | **< .001** |
| *3.1. Successful learning processes in general* | *1* | *0.50%* | *18* | *0.57%* | *19* | *0.57%* | *0.019* | *> .999** |
| *3.2. Successfully learning alone/independently* | *8* | *3.96%* | *330* | *10.45%* | *338* | *10.06%* | *8.843* | *.003* |
| 3.2.1 Successfully learning alone/independently in general | 2 | 0.99% | 171 | 5.42% | 173 | 5.15% | - | - |
| 3.2.2. Being able to set priorities while learning | 1 | 0.50% | 30 | 0.95% | 31 | 0.92% | - | - |
| 3.2.3. Being able to learn at one’s own pace | 5 | 2.48% | 129 | 4.09% | 134 | 3.99% | - | - |
| *3.3. Being able to concentrate/avoiding distractions* | *3* | *1.49%* | *200* | *6.34%* | *203* | *6.04%* | *7.865* | *.005* |
| *3.4. Motivational and volitional success* | *3* | *1.49%* | *108* | *3.42%* | *111* | *3.30%* | *2.227* | *.136* |
| 3.4.1. Being motivated | 0 | 0.00% | 37 | 1.17% | 37 | 1.10% | - | - |
| 3.4.2. Being engaged in learning | 0 | 0.00% | 13 | 0.41% | 13 | 0.39% | - | - |
| 3.4.3. Finding joy in learning | 1 | 0.50% | 24 | 0.76% | 25 | 0.74% | - | - |
| 3.4.4. Being (self-)disciplined | 2 | 0.99% | 34 | 1.08% | 36 | 1.07% | - | - |
| *3.5. Being organized* | *4* | *1.98%* | *544* | *17.23%* | *548* | *16.31%* | *32.345* | *<.001* |
| 3.5.1. Being organized in general | 3 | 1.49% | 61 | 1.93% | 64 | 1.91% | - | - |
| 3.5.2. Successfully upholding a daily structure | 0 | 0.00% | 8 | 0.25% | 8 | 0.24% | - | - |
| 3.5.3. Successfully managing tasks and time | 1 | 0.50% | 321 | 10.17% | 322 | 9.59% | - | - |
| 3.5.4. Successfully keeping track of tasks to be done | 0 | 0.00% | 10 | 0.32% | 10 | 0.30% | - | - |
| 3.5.5. Successfully adhering to deadlines | 0 | 0.00% | 144 | 4.56% | 144 | 4.29% | - | - |
| **4. Contextual conditions** | **1** | **0.50%** | **155** | **4.91%** | **156** | **4.64%** | **8.355** | **.004** |
| *4.1. Having good learning materials* | *0* | *0.00%* | *4* | *0.13%* | *4* | *0.12%* | *0.256* | *> .999** |
| *4.2. Good learning environment* | *1* | *0.50%* | *59* | *1.87%* | *60* | *1.79%* | *2.042* | *.264** |
| *4.3. Successful digital learning* | *0* | *0.00%* | *92* | *2.91%* | *92* | *2.74%* | *6.052* | *.014* |
| 4.3.1. Successful digitally mediated learning | 0 | 0.00% | 23 | 0.73% | 23 | 0.68% | - | - |
| 4.3.2. Successful online communication | 0 | 0.00% | 19 | 0.60% | 19 | 0.57% | - | - |
| 4.3.2.1. Receiving and handing in/doing (online) assignments | 0 | 0.00% | 16 | 0.51% | 16 | 0.48% | - | - |
| 4.3.2.2. Successfully working with communication platforms | 0 | 0.00% | 3 | 0.10% | 3 | 0.09% | - | - |
| 4.3.3. Successfully working on the computer | 0 | 0.00% | 49 | 1.55% | 49 | 1.46% | - | - |
| 4.3.3.1. Successfully working on the computer in general | 0 | 0.00% | 37 | 1.17% | 37 | 1.10% | - | - |
| 4.3.3.2. Advantages when working at the computer | 0 | 0.00% | 9 | 0.29% | 9 | 0.27% | - | - |
| 4.3.3.3. Liking to work on the computer | 0 | 0.00% | 3 | 0.10% | 3 | 0.09% | - | - |
| 4.3.4. Having good equipment | 0 | 0.00% | 1 | 0.03% | 1 | 0.03% | - | - |
| **5. Well-being** | **1** | **0.50%** | **35** | **1.11%** | **36** | **1.07%** | **0.674** | **.722*** |
| *5.1. Successfully keeping up psychological well-being* | *1* | *0.50%* | *33* | *1.05%* | *34* | *1.01%* | *0.574* | *.720** |
| *5.2. Successfully keeping up physical well-being* | *0* | *0.00%* | *2* | *0.06%* | *2* | *0.06%* | *0.128* | *> .999** |
| **6. Everything is going well** | **1** | **0.50%** | **466** | **14.76%** | **467** | **13.90%** | **32.278** | **< .001** |
| **7. Nothing is going well** | **107** | **52.97%** | **10** | **0.32%** | **117** | **3.48%** | **1565.641** | **< .001** |
| **8. Residual Category** | **29** | **14.36%** | **124** | **3.93%** | **153** | **4.55%** | **47.494** | **< .001** |
| Sum | 202 | 100% | 3157 | 100% | 3359 | 100% |  |  |
| N = Documents | 235 |  | 2417 |  | 2652 |  |  |  |
| *Note.* Percentages for the separate groups were calculated by relating the number of coded segments in a specific category to the number of coded segments in the group overall. Total percentages were related to the overall number of coded segments,  *In case of more than 20% of the cells having and expected absolute value < 5 Fisher’s Exact Test (2-sided) has been reported. | | | | | | | | |
