# Supplementary material for: Learning during COVID-19: the role of self-regulated learning, motivation, and procrastination for perceived competence
Source: Z Erziehwiss. 2021 Mar 4;24(2):393–418. doi: 10.1007/s11618-021-01002-x (PMC7931168; doi:10.1007/s11618-021-01002-x)
Supplement: Supplementary file 3 — Table III. Quantitative summary of Question 3: “With what could you currently use some help?” [file 11618_2021_1002_MOESM3_ESM.docx]

| Table III  *Quantitative summary of Question 3: “With what could you currently use some help?”* | | | | | | | | |
| --- | --- | --- | --- | --- | --- | --- | --- | --- |
|  | **low competence** | | **high competence** | | **total** | | ***χ²*** | ***p*** |
|  | sum | rel. % | sum | rel. % | sum | rel. % |  |  |
| **1. Contact with others** | **43** | **13.52%** | **114** | **5.25%** | **157** | **6.31%** | **32.107** | **< .001** |
| *1.1. Wanting contact with/support from guardians/family* | *0* | *0.00%* | *2* | *0.09%* | *2* | *0.08%* | *0.293* | *> .999** |
| *1.2 Wanting contact with/support from peers* | *1* | *0.31%* | *3* | *0.14%* | *4* | *0.16%* | *0.537* | *.421* |
| *1.3. Wanting contact with/support from teachers* | *42* | *13.21%* | *109* | *5.02%* | *151* | *6.07%* | *32.623* | *< .001* |
| 1.3.1 Wanting contact with/support from teachers in general | 6 | 1.89% | 14 | 0.64% | 20 | 0.80% | - | - |
| 1.3.2. Needing clear instructions/help understanding assignments | 8 | 2.52% | 28 | 1.29% | 36 | 1.45% | - | - |
| 1.3.3. Having a teacher explaining things | 20 | 6.29% | 41 | 1.89% | 61 | 2.45% | - | - |
| 1.3.4. Needing the opportunity to ask questions | 4 | 1.26% | 19 | 0.88% | 23 | 0.92% | - | - |
| 1.3.5. Getting (timely) feedback | 1 | 0.31% | 7 | 0.32% | 8 | 0.32% | - | - |
| 1.3.6. Wanting teachers to have empathy/understanding for the situation | 3 | 0.94% | 0 | 0.00% | 3 | 0.12% | - | - |
| **2. Learning outcomes** | **145** | **45.60%** | **851** | **39.20%** | **996** | **40.02%** | **4.732** | **.030** |
| *2.1. Wanting support in completing assignments* | *1* | *0.31%* | *0* | *0.00%* | *1* | *0.04%* | *6.830* | *.128* |
| *2.2. Wanting support in learning and/or understanding (new) material* | *17* | *5.35%* | *112* | *5.16%* | *129* | *5.18%* | *0.020* | *.888* |
| *2.3. Wanting support in preparing for the final exams (Matura)* | *6* | *1.89%* | *13* | *0.60%* | *19* | *0.76%* | *6.074* | *.026* |
| *2.4. Wanting support in specific subjects or tasks* | *121* | *38.05%* | *726* | *33.44%* | *847* | *34.03%* | *2.625* | *.105* |
| 2.4.1. Wanting support in specific subjects or tasks in general | 15 | 4.72% | 79 | 3.64% | 94 | 3.78% | - | - |
| 2.4.2. Wanting support in working for (perceived) boring/unimportant subjects | 0 | 0.00% | 2 | 0.09% | 2 | 0.08% | - | - |
| 2.4.3. Wanting support in Mathematics | 57 | 17.92% | 309 | 14.23% | 366 | 14.70% | - | - |
| 2.4.4. Wanting support in German | 9 | 2.83% | 74 | 3.41% | 83 | 3.33% | - | - |
| 2.4.5. Wanting support in English | 9 | 2.83% | 122 | 5.62% | 131 | 5.26% | - | - |
| 2.4.6. Wanting support in Physics | 5 | 1.57% | 23 | 1.06% | 28 | 1.12% | - | - |
| 2.4.7. Wanting support in economical subjects | 8 | 2.52% | 29 | 1.34% | 37 | 1.49% | - | - |
| 2.4.8. Wanting support in other subjects | 18 | 5.66% | 88 | 4.05% | 106 | 4.26% | - | - |
| **3. Learning process** | **60** | **18.87%** | **42** | **1.93%** | **102** | **4.10%** | **202.369** | **< .001** |
| *3.1. Wanting support in the learning process in general* | *3* | *0.94%* | *0* | *0.00%* | *3* | *0.12%* | *20.442* | *< .002** |
| *3.2. Wanting support in learning independently* | *1* | *0.31%* | *0* | *0.00%* | *1* | *0.04%* | *6.830* | *.128** |
| *3.3. Wanting support in learning to concentrate/avoid distractions* | *6* | *1.89%* | *7* | *0.32%* | *13* | *0.52%* | *13.064* | *.003** |
| *3.4. Wanting support with motivational and volitional challenge* | *23* | *7.23%* | *9* | *0.41%* | *32* | *1.29%* | *101.599* | *< .001** |
| 3.4.1. Wanting support in building/maintaining motivation | 17 | 5.35% | 8 | 0.37% | 25 | 1.00% | - | - |
| 3.4.2. Wanting support in building/maintaining (self-)discipline | 6 | 1.89% | 1 | 0.05% | 7 | 0.28% | - | - |
| *3.5. Wanting support with (self-)organization* | *27* | *8.49%* | *26* | *1.20%* | *53* | *2.13%* | *70.789* | *< .001* |
| 3.5.1. Wanting support with (self-)organization in general | 5 | 1.57% | 7 | 0.32% | 12 | 0.48% | - | - |
| 3.5.2. Wanting support in building/maintaining a daily structure | 5 | 1.57% | 0 | 0.00% | 5 | 0.20% | - | - |
| 3.5.3. Wanting support in managing tasks and time | 11 | 3.46% | 13 | 0.60% | 24 | 0.96% | - | - |
| 3.5.4. Wanting support in keeping track of tasks to be done | 6 | 1.89% | 5 | 0.23% | 11 | 0.44% | - | - |
| 3.5.5. Wanting support in adhering to deadlines | 0 | 0.00% | 1 | 0.05% | 1 | 0.04% | - | - |
| **4. Contextual conditions** | **13** | **4.09%** | **72** | **3.32%** | **85** | **3.42%** | **0.501** | **.479** |
| *4.1. Wanting good learning materials* | *1* | *0.31%* | *5* | *0.23%* | *6* | *0.24%* | *0.082* | *.560** |
| *4.2. Wishing for a supportive learning environment* | *2* | *0.63%* | *0* | *0.00%* | *2* | *0.08%* | *13.665* | *.016** |
| *4.3. Wanting support in dealing with school-related requirements* | *8* | *2.52%* | *18* | *0.83%* | *26* | *1.04%* | *7.633* | *.013** |
| 4.3.1. Wishing for support in dealing with too high school-related requirements | 8 | 2.52% | 17 | 0.78% | 25 | 1.00% | - | - |
| 4.3.1.1. Wishing for better agreement/coordination between the teachers | 0 | 0.00% | 1 | 0.05% | 1 | 0.04% | - | - |
| 4.3.1.2. Wishing for more time for finishing assignments | 1 | 0.31% | 4 | 0.18% | 5 | 0.20% | - | - |
| 4.3.1.3. Wishing for fewer assignments | 7 | 2.20% | 12 | 0.55% | 19 | 0.76% | - | - |
| 4.3.2. Wishing for more learning material to further interest/talents | 0 | 0.00% | 1 | 0.05% | 1 | 0.04% | - | - |
| *4.4. Wishing for support in dealing with distance learning setting* | *2* | *0.63%* | *49* | *2.26%* | *51* | *2.05%* | *3.663* | *.056* |
| 4.4.1. Wishing for teachers being more tech savvy | 1 | 0.31% | 1 | 0.05% | 2 | 0.08% | - | - |
| 4.4.2. Wishing for assistance in receiving and handing in assigments | 1 | 0.31% | 9 | 0.41% | 10 | 0.40% | - | - |
| 4.4.4. Wanting support in working with communication platforms | 0 | 0.00% | 5 | 0.23% | 5 | 0.20% | - | - |
| 4.4.4.1. Wishing for unified communication | 0 | 0.00% | 4 | 0.18% | 4 | 0.16% | - | - |
| 4.4.4.2. Wanting support when communication platforms don’t work | 0 | 0.00% | 1 | 0.05% | 1 | 0.04% | - | - |
| 4.4.3. Wanting support in digitally mediated teaching & learning | 0 | 0.00% | 1 | 0.05% | 1 | 0.04% | - | - |
| 4.4.5. Wanting support in working on the computer | 0 | 0.00% | 23 | 1.06% | 23 | 0.92% | - | - |
| 4.4.6. Wanting support in dealing with technical equipment | 0 | 0.00% | 11 | 0.51% | 11 | 0.44% | - | - |
| 4.4.6.1 Wanting support in dealing with technical equipment - not specified | 0 | 0.00% | 3 | 0.14% | 3 | 0.12% | - | - |
| 4.4.6.1. Needing the necessary equipment | 0 | 0.00% | 4 | 0.18% | 4 | 0.16% | - | - |
| 4.4.6.2. Needing a better internet connection/(wireless) network | 0 | 0.00% | 4 | 0.18% | 4 | 0.16% | - | - |
| **5. Well-being** | **5** | **1.57%** | **0** | **0.00%** | **5** | **0.20%** | **34.204** | **< .001*** |
| **6. Support is needed in everything** | **33** | **10.38%** | **9** | **0.41%** | **42** | **1.69%** | **165.956** | **< .001** |
| **7. No further support necessary** | **7** | **2.20%** | **1012** | **46.61%** | **1019** | **40.94%** | **226.279** | **< .001** |
| **8. Residual Category** | **12** | **3.77%** | **70** | **3.22%** | **82** | **3.29%** | **0.263** | **.608** |
| Sum | 318 | 100% | 2171 | 100% | 2489 | 100% |  |  |
| N = Documents | 235 |  | 2417 |  | 2652 |  |  |  |
