# Supplementary material for: Learning during COVID-19: the role of self-regulated learning, motivation, and procrastination for perceived competence
Source: Z Erziehwiss. 2021 Mar 4;24(2):393–418. doi: 10.1007/s11618-021-01002-x (PMC7931168; doi:10.1007/s11618-021-01002-x)
Supplement: Supplementary file 4 — Table IV. Category system for Question 1: “What do you currently find especially hard when studying?” [file 11618_2021_1002_MOESM4_ESM.docx]

## Table IV

## *Category system for Question 1: “What do you currently find especially hard when studying?”*

| **Category** | | **Coding rules** | | **Examples** | |
| --- | --- | --- | --- | --- | --- |
| **1. Contact with others** | |  | |  | |
| - 1. *Lack of contact/support with/from others in general* | | Statements about the lack of contact with or support from others without further specification. | | „Die Face to Face Konversation fehlt.“ | |
| *1.2. Lack of contact/support with/from guardians/family* | | Statements referring to having no or too little contact with or support from family members or caregivers. | | “Keine Freunde, Bekannte oder Familie treffen.“ | |
| *1.3. Lack of contact/support with/from peers* | | Statements referring to having no or too little contact with peers, both in their free time and in relation to learning together. Statement regarding the lack of mutual support or difficulties working with peers | | “Dass ich mit niemanden zB eine Teamarbeit machen kann und meine Freunde nicht sehe.“ | |
| *1.4. Lack of contact/support with/from teachers* | |  | |  | |
| 1.4.1. Lack of contact/support with/from teachers in general | | Statements referring to having no or too little contact with or support from teachers in general (e.g. no face to face lessons, no communication with teachers). | | “der lehrerkontakt (frontalunterricht fehlt mir)“ | |
| 1.4.2. Unclear/incomprehensible tasks | | Statements about getting unclear or incomprehensible instructions or assignments. | | „Manchmal werden von Lehrern Arbeitsaufträge nicht genau aufgegeben sodass man sich nicht auskennt was man jetzt machen soll“ | |
| 1.4.3. Too few explanations | | Statements about the lack of explanations on assignments or subject matters. | | „In der Schule erklären Professoren die Aufgaben, aber zu Hause muss man sich die Aufgaben selber erklären.“ | |
| 1.4.4. Difficulties getting answers to questions | | Statements about teachers being difficult to contact for answers to questions, delayed answers or not getting answers at all. | | „Wenn ich mich bei einer Aufgabe nicht auskennen sollte, bekomme ich von den Professoren natürlich nicht sofort eine Rückmeldung oder Erklärung. Ärgerlich ist, dass man für eine Rückmeldung einige Tage warten muss.“ | |
| 1.4.5. Too little/late feedback | | Statements referring to teachers giving no or too little or delayed feedback. | | „Kein direktes Feedback vom Lehrer“ | |
| 1.4.6. Lack of understanding/empathy for the situation | | Statements about teachers not showing empathy or understanding for the difficulty of the situation. | | „Das manche Lehrer kein Verständnis für schwierige Situationen haben“ | |
| **2. Learning outcomes** | |  | |  | |
| *2.1. Challenges in completing assignments* | |  | |  | |
| 2.1.1. Challenges in completing assignments in general | | Statements about difficulties in completing assignments. Also, statements about taking too long in completing assignments or taking longer than in a regular school setting.  Statements referring to working on assignments or completing assignments in a diligent or accurate manner (e.g. taking care to write in pretty handwriting, taking care of doing tasks correctly) are coded in 🡪 Challenges in completing assignments diligently/accurately diligently/accurately.  Statements referring to working effectively or regarding productivity are coded in 🡪 Challenges in completing assignments effectively/productively effectively/productively. | | „Alle Arbeitsaufträge fertig zu machen“ | |
| 2.1.2. Challenges in completing assignments diligently/accurately | | Statements referring to working on assignments or completing assignments in a diligent or accurate manner (e.g. taking care to write in pretty handwriting, taking care of doing tasks correctly). | | „Alles ordentlich durchzuarbeiten.“ | |
| 2.1.3. Challenges in completing assignments effectively/productively | | Statements referring to working effectively or regarding productivity. | | „effektives Arbeiten und Vorankommen“ | |
| *2.2. Challenges when learning and/or understanding (new) material* | | Difficulties in learning or comprehending new or complicated material or in rehearsing old material.  Statements referring to independent learning of (new) material were also coded under 🡪 3.2. Challenges when learning alone/independently.  Statements referring to teachers not explaining (new) material were also coded in 🡪 1.4.2. Too few explanations.  Statements about learning (new) material in a specific subject were also coded in 🡪 2.4. Challenges regarding specific subjects or tasks in the respective subcategory. | | „Dinge die wir noch nicht gemacht haben“ | |
| *2.3. Challenges regarding preparation for the final exams (Matura)* | |  | | „Vorbereitung auf Matura“ | |
| *2.4. Challenges regarding specific subjects/ tasks/assignments* | | Statements about needing support referring to specific subjects, final exams or subjects, that are perceived as boring or unimportant are coded in the respective subcategory (subjects that were mentioned more than 20 times were kept in separate categories). | |  | |
| 2.4.1 Challenges regarding specific subjects/tasks/assignments in general | | Statements about preparation for the final exams (Matura) being challenging. | | „Verbesserungen zu machen“ | |
| 2.4.2. Challenges in working for (perceived) boring/unimportant subjects | | Statements about tasks, assignments or activities related to school or learning that are perceived as particularly difficult.  Statements referring to specific subjects, final exams or subjects, that are perceived as boring or unimportant are also coded in the respective subcategory. | | „Dass viele Aufhaben sinnlos sind“ | |
| 2.4.3. Challenges in Mathematics | | Statements about the subject Mathematics being challenging.  Statements referring to challenges in learning or understanding (new) material in Mathematics are also coded in🡪 2.2. Challenges when learning and/or understanding (new) material.  Statements referring to getting too few explanations from teachers in Mathematics are also coded in 🡪 1.4.2. Too few explanations. | | „manchmal Mathe wenn was neues ist“ | |
| 2.4.4. Challenges in German | | Statements about the subject German being challenging.  Statements referring to challenges in learning or understanding (new) material in German are also coded in 🡪 2.2. Challenges when learning and/or understanding (new) material.  Statements referring to getting too few explanations from teachers in German are also coded in 🡪 1.4.2. Too few explanations. | | „Deutsch ist am meisten schwierig.“ | |
| 2.4.5. Challenges in English | | Statements about the subject English being challenging.  Statements referring to challenges in learning or understanding (new) material in English are also coded in 🡪 2.2. Challenges when learning and/or understanding (new) material.  Statements referring to getting too few explanations from teachers in English are also coded in 🡪 1.4.2. Too few explanations. | | „Die Cyber Homework in Englisch“ | |
| 2.4.6. Challenges in Other subjects | | Statements about other subjects being challenging (e.g. Latin, Music, Physics, ...).  Statements referring to challenges in learning or understanding (new) material in other subjects are also coded in 🡪 2.2. Challenges when learning and/or understanding (new) material.  Statements referring to getting too few explanations from teachers in other subjects are also coded in 🡪 1.4.2. Too few explanations. | | „Fächer wie Unternehmensrechnung“ | |
| **3. Learning process** | |  | |  | |
| *3.1. Challenges regarding the learning process in general* | | Statements about difficulties regarding the learning process that could not be further categorized (e.g., “learning”). | | “Das lernen” | |
| *3.2. Challenges when learning alone/independently* | | Statements about having to learn alone or challenges when learning independently.  Statements referring to the lack of support or not getting help were coded in 🡪 1. Contact with others in the respective subcategories. | | „das eigenständige Lernen“ | |
| *3.3. Difficulties concentrating/avoiding distractions* | | Statements about having difficulties to concentrate and not getting distracted.  Statements referring to a distracting learning environment (e.g. family at home) were coded in 🡪 4.2. Challenging learning environment. | | „Konzentration“  „sich länger zu konzentrieren“ | |
| *3.4. Motivational and volitional challenges* | |  | |  | |
| 3.3.1. Lack of motivation | | Statements about difficulties in motivating oneself to learn or in starting with a task. | | „Mich aufzuraffen mal irgendetwas anzufangen.“ | |
| 3.3.2. Lack of joy in learning | | Statements about the lack of joy or having no interest in or fun while learning. | | „kein Spaß am Lernen habe.“ | |
| 3.3.3. Lack of (self-)discipline | | Statements about having difficulties following through with a plan. | | „Mich zu überwinden alles zu machen und besonders diszipliniert zu sein“ | |
| *3.4. Difficulties in (self-)organization* | |  | |  | |
| 3.4.1. Difficulties in (self-)organization in general | | Statement referring to difficulties in structuring tasks and organizing oneself in general.  Statements about difficulties in keeping a daily structure (🡪 3.4.2. Difficulties keeping daily structure), managing time and planning tasks (🡪 3.4.3. Difficulties in managing tasks and time), keeping track of tasks to be done (🡪 3.4.4. difficulties keeping track of tasks to be done) and adhering to deadlines (🡪 3.4.5. Difficulties adhering to deadlines) were coded in the respective subcategory. | | “Schlechte Organisation und Struktur” | |
| 3.4.2. Difficulties due to the lack of a daily structure | | Statements referring to difficulties in keeping up a daily routine or structure or about having no given timetable or schedule. | | „Keinen normalen Ablauf wie sonst zu haben.“ | |
| 3.4.3. Difficulties in managing tasks and time | | Statements about challenges in managing time well and/or creating a learning plan and/or ill use of time (e.g. starting to work at the end of the day). | | „Arbeitsaufträge zeitlich zu organisieren” | |
| 3.4.4. Difficulties keeping track of tasks to be done | | Statements about difficulties in keeping track of the tasks to be done. May also be due to receiving new assignments during the week or due to having too many different deadlines.  Statements about difficulties adhering to deadlines due to keeping track of different deadlines were also coded in 🡪 3.4.5. Difficulties adhering to deadlines.  Statements referring to difficulties keeping track of tasks because of the variety of platforms that are used for communication by teachers were also coded in 🡪 4.4.4.1. Too many different communication platforms. | | „Einen Überblick über alle Aufgaben zu haben, wenn alle Aufgaben gleichzeitig geschickt werden“ | |
| 3.4.5. Difficulties adhering to deadlines | | Statements about difficulties adhering to deadlines. Can also be due having multiple deadlines in different subjects. | | „pünktlich die Aufgaben einzureichen;“ | |
| **4. Contextual conditions** | |  | |  | |
| *4.1. Challenges due to poor learning materials* | | Statements about difficulties in learning due to a lack of (good) learning materials. | | „Das manchmal das Material zu neuen Themen eher suboptimal ist (z.B. unvollständige Youtube-Videos)“ | |
| *4.2. Challenging learning environment* | | Statements referring to difficulties due to a challenging learning environment (e.g., too much noise at home or distractions). | | “Einen ruhigen Ort zum Lernen zu finden“ | |
| *4.3. Challenging school-related requirements* | |  | |  | |
| 4.3.1. Too high school requirements | | Statements about difficulties due to too high school requirements in general. | | „zu lange Unterrichtseinheiten“ | |
| 4.3.1.1. ... due to too little coordination between the teachers | | Statements about challenges arising because teachers don’t coordinate their assignments.  Statements about teachers using too many different platforms to communicate were coded in 🡪 4.4.4.1. Too many different communication platforms. | | „Keine Abstimmung der Lehrer untereinander über Lehrmenge, Projekte und Abgabefristen“  „Die Kommunikation zwischen den Professoren.“ | |
| 4.3.1.2. ... due to too much time pressure | | Statements about having too much time pressure/too little time for finishing assignments. | | „die knappe Zeit die zur Verfügung steht für die Arbeitsaufträge“ | |
| 4.3.1.3. ... due to too many/too demanding tasks | | Statements about getting too many or too demanding assignments. | | „Es sind zu viele Aufgaben“ | |
| 4.3.2. Too low school-related requirements | | Statements about being challenged too little when learning. | | „Leistungsfoederung“ | |
| *4.4. Challenges due to the digital learning setting* | | Statements about distance or digital learning being challenging, without further specification. | | „Lernen auf Distanz“ | |
| 4.4.1. Teachers are not familiar with the necessary technology | | Statements about teachers having not enough technical knowledge to support digital learning. | | „Dass die Lehrer sich nicht mit der Technik auskennen“ | |
| 4.4.2. Receiving and handing in/doing (online) assignments | | Statements about getting too many e-mails and about difficulties in sending or receiving assignments (also digitally) and doing online assignments. | | „Sachen auf seltsame Platformen hochzuladen“ | |
| 4.4.3. Challenges due to digitally mediated teaching & learning | | Statements that it is sometimes difficult to convey or explain content via digital media. | | „Komplexe Themen benötigen oft viieeel Zeit und erklärungsbedarf, was über Emailverkehr nicht wirklich umsetzbar ist.“ | |
| 4.4.4. Challenges working with communication platforms | |  | |  | |
| 4.4.4.1. Too many different communication platforms | | Statements about teachers using too many different platforms to communicate or send and receive assignments. | | „Das jeweilige Professoren nicht über eine Platform kommunizieren, sondern über mehrere.“ | |
| 4.4.4.2. Communication platforms don’t work | | Statements that the used communication platforms don’t work. | | „TSN und Moodle schwer fehlerhaft, manchmal werden E-Mails nicht empfangen“ | |
| 4.4.5. Challenges when working on the computer | | Statements about not being able to handle the software or hardware used, or not handling it well enough. | | „Wenn ich mich am Computer nicht auskenne.“ | |
| 4.4.6. Spending a lot of time in front of the computer | | Statements about spending too much time in front of the computer screen. | | „Ständig auf einen Bildschirm zu blicken (ich habe trotz Pausen ständige Kopfschmerzen davon)“ | |
| 4.4.7. Difficulties with the necessary equipment | | Statements about difficulties with the technical equipment. | |  | |
| 4.4.7.1. Technical equipment doesn’t work | | Statements that the technical equipment doesn’t work. | | „Wenn der Drucker nicht funktioniert und wir dann keine gescheiten Kopien haben!“ | |
| 4.4.7.2. Not having the necessary equipment | | Statements about difficulties due to not having access to the necessary equipment or software or having to share the equipment with others. | | „ich teile mir mit meiner kleinen Schwester (2. Klasse Mittelschule) einen Laptop und es ist manchmal schwierig, seine Aufgaben online zu machen, da die andere den Laptop auch braucht.“ | |
| 4.4.7.3. Problems with the Internet connection or (wireless) network | | Statements about difficulties arising due to a slow or Internet connection or (wireless) network as well as not having an Internet connection at all. | | „Eine sehr langsame Internetverbindung.“ | |
| *4.5. Additional burdens due to other obligations or chores* | | Statements about having other obligations besides school (e.g. chores around the house, babysitting siblings). | | „Wenig Zeit für Aufgaben weil ich mich um Geschwister kümmern muss“ | |
| **5. Well-being** | |  | |  | |
| *5.1. Psychological challenges* | |  | |  | |
| 5.1.1. Helplessness/anxiety/stress | | Statements about feeling helpless or stressed or being anxious or overwhelmed. | | „Dem Druck der Schule standhalten“ | |
| 5.1.2. Dealing with uncertainty | | Statements about having to deal with the uncertainty of the situation. | | „nicht zu wissen wann Prüfungen stattfinden werden“ | |
| 5.1.3. Learning-life balance | | Statements that it is difficult to maintain a balance between learning and other school related tasks and other aspects of life. | | „Balance zwischen Interessen der Familie und der Schule“ | |
| 5.1.4. Family difficulties | | Statements referring to family difficulties (e.g. arguments with parents or siblings). | | „Streitereien mit meinen Eltern“ | |
| *5.2. Physical challenges* | | Statements referring to the situation being physically challenging (e.g. not enough time or space to work out). | | „kaum sport und bewegung“ | |
| **6. Everything is challenging** | | Statements that everything feels challenging right now. | | „Alles Ok“ | |
| **7. Nothing is challenging** | | Statements that everything is fine, and nothing is challenging. | | „Alles ist schwierig“ | |
| **8. Residual Category** | | Non-content-bearing statements, non-topic-related answers, answers with insufficient specification | | “Das sich der Staat noch immer einmischt!!!!“ | |
