# Supplementary material for: Learning during COVID-19: the role of self-regulated learning, motivation, and procrastination for perceived competence
Source: Z Erziehwiss. 2021 Mar 4;24(2):393–418. doi: 10.1007/s11618-021-01002-x (PMC7931168; doi:10.1007/s11618-021-01002-x)
Supplement: Supplementary file 5 — Table V. Category system for Question 2: “What parts of studying are currently going particularly well?” [file 11618_2021_1002_MOESM5_ESM.docx]

## Table V

## *Category system for Question 2: “What parts of studying are currently going particularly well?”*

| **Category** | **Coding rules** | **Examples** | |
| --- | --- | --- | --- |
| **1. Contact with others** |  |  | |
| *1.1. Receiving support from/keeping in contact with others in general* | Statements about getting support from or keeping in contact with others without further specification. | „Aufgaben zu verstehen, weil persönliche Hilfe“ | |
| *1.2. Receiving support from/keeping in contact with guardians/family* | Statements referring getting support from or keeping in contact with family members or caregivers. | „Hilfe meine Geschwister“ | |
| *1.3. Receiving support from/keeping in contact with peers* | Statements referring getting support from or keeping in contact with peers. | „Dass ich über Facetime mit meiner Freundin lernen kann“ | |
| *1.4. Receiving support from/keeping in contact with teachers* |  |  | |
| 1.4.1. Receiving support from/keeping in contact with teachers in general | Statements referring getting support from or keeping in contact with teachers in general (e.g. communicating with teachers). | “Wenn ich mich wo nicht auskenne, hilft mir mein Lehrer jetzt besser als in der Schule.“ | |
| 1.4.2. Teachers give clear and clear/comprehensible instructions | Statements referring to teachers giving clear and comprehensible instructions. | „Die Aufgaben die wir bekommen, sind sehr verständlich und nicht schwer und es ist immer beschrieben.“ | |
| 1.4.3. Teachers give comprehensive explanations | Statements that teacher give comprehensive explanations that help understanding learning material. | „da wir alles sehr genau erklärt bekommen.“ | |
| 1.4.4. Teachers answering questions (in time) | Statements about teachers being available should questions arise. | „wenn ich Hilfe brauche kann ich meine Lehrern fragen und es ist jeden Tag um 13 und 14 Uhr wenn man fragen hat.“ | |
| 1.4.5. Teachers giving (timely) feedback | Statements about teachers giving (timely) feedback. | „Kein direktes Feedback vom Lehrer“ | |
| **2. Learning outcomes** |  |  | |
| *2.1. Success in achieving learning outcomes in general* | Statements about achieving learning outcomes in general (e.g. finding solutions, feeling that they succeed). | „Ich kann es danach“ | |
| *2.2. Success in understanding assignments* | Statements about understanding assignments and tasks. | „Das verstehen der Aufgaben.“ | |
| *2.3. Success in completing assignments* |  |  | |
| 2.3.1. Success in completing assignments in general | Statements about (successfully) completing the given assignments.  Statements referring to doing assignments 🡪 2.3.2. ... diligently/accurately) or 🡪 2.3.3. ... effectively/productively) were coded in the respective subcategory.  Statements referring to handing in assignments on time were coded in 🡪 3.5.5. Successfully adhering to deadlines | „Abarbeitung aller Aufgaben“ | |
| 2.3.2. ... diligently/accurately | Statements referring to doing the assignments diligently/accurately and/or correctly. | “Die Arbeitsaufträge genau und sorgfältig zu machen.“ | |
| 2.3.3. ... effectively/productively | Statements referring to working effectively or regarding productivity. | „Jeder Arbeitsauftrag wird so schnell wie möglich gemacht“ | |
| *2.4. Successfully learning (new) material* | Statements referring to understanding (new) learning material, also due learning more thoroughly.  Statements referring to independent learning of (new) material were also coded under 🡪 3.2. Successfully learning alone/independently.  Statements referring to successfully understanding (new) learning material due to being able to learn at one’s own pace were also coded in 🡪 3.2.3. Being able to learn at one’s own pace.  Statements referring to teachers not explaining (new) material were also coded in 🡪 1.4.3 Teachers are giving comprehensive explanations.  Statements about learning (new) material in a specific subject were also coded in 🡪 2.6. Successful learning outcomes in specific subjects/tasks/assignments in the respective subcategory. | „intensiveres Beschäftigen mit dem Lernstoff und dadurch merkt man sich mehr als bei regulärem Unterricht.“ | |
| *2.5. Successfully preparing for the final exams (Matura)* | Statements regarding successful preparation for the final exams (Matura) being challenging. | „VWA“ | |
| *2.6. Successful learning outcomes in/with specific subjects/tasks/assignments* |  |  | |
| 2.6.1. Successful learning outcomes in specific subjects/tasks/assignments in general | Statements about succeeding in tasks, assignments or activities related to school or learning that are not related to a specific subject.  Statements referring to specific subjects, final exams or subjects were coded in the respective subcategory (subjects that were mentioned more than 20 times were kept in separate categories). | „Zusammenfassungen zum Stoff zu schreiben“  „Mit Portfolio und Präsentationen“ | |
| 2.6.2. Achieving learning outcomes in Mathematics | Statements about being successful in Mathematics.  Statements referring to successfully learning or understanding (new) material in Mathematics were also coded in 🡪 2.4. Successfully learning (new) material. | „Mathematik gelingt mir zurzeit sehr gut“ | |
| 2.6.3. Achieving learning outcomes in German | Statements about being successful in German.  Statements referring to successfully learning or understanding (new) material in German were also coded in 🡪 2.4. Successfully learning (new) material. | „Deutsch“ | |
| 2.6.4. Achieving learning outcomes in English | Statements about being successful in English.  Statements referring to successfully learning or understanding (new) material in English were also coded in 🡪 2.4. Successfully learning (new) material. | “ich lerne sehr gut und leicht Englisch zu Hause“ | |
| 2.6.5. Achieving learning outcomes in other subjects | Statements about being successful in other subjects.  Statements referring to successfully learning or understanding (new) material in other subjects were also coded in 🡪 2.4. Successfully learning (new) material. | “Chemie” | |
| *2.7. Getting good/better feedback/grades* | Statements about getting positive feedback and/or better grades in home-learning than in regular school setting. | „positive Rückmeldungen von meinen Lehrern“  „habe viel bessere Noten“ | |
| **3. Learning Process** |  |  | |
| *3.1. Successful learning processes in general* | Statements about learning successfully in general. | “Mit geht es sehr gut mit dem Lernen.” | |
| *3.2. Successfully learning alone/independently* |  |  | |
| 3.2.1 Successfully learning alone/independently – not specified | Statements about being successful in learning independently/self-responsible and/or alone. | „selbstständiges lernen“ | |
| 3.2.2. Being able to set priorities while learning | Statements about being able to dive deeper in subjects that are more interesting or need more attention. | “Fächer für die ich mehr Zeit brauche kann ich sie mir nehmen. Und bei anderen wo ich sie nicht brauche kann ich mir Zeit sparen.” | |
| 3.2.3. Being able to learn at your own pace | Statements about having more time to deal with assignments or subjects. | „in meiner Geschwindigkeit die Sachen erledigen kann“ | |
| *3.3. Being able to concentrate/avoiding distractions* | Statements about being able to concentrate on the task at hand and successfully deflecting distractions.  Statements specifically referring to being able to better concentrate due to a less distracting learning environment were (also) coded in 🡪 4.2. Good learning environment. | „ich kann mich besser konzentrieren als in der Schule“ | |
| 3.4. Motivational and volitional success |  |  | |
| 3.4.1. Being motivated | Statements about being interested in the subject at hand, being motivated to and getting started to/with learn. | „Ich bin extremst motiviert zu lernen“ |  |
| 3.4.2. Being engaged in learning | Statements about being engaged in the learning process (unlike in school, where one can drift off). | „Ich bin nicht mehr der Gefahr ausgesetzt, wie in der Schule den Lehrer reden zu lassen und nichts zu tun.“ |  |
| 3.4.3. Finding joy in learning | Statements about finding joy in learning. | „In vielen Fächern habe ich gerade viel mehr Freude daran Neues zu lernen, als unter der regulären Schulzeit.“ |  |
| 3.4.4. Being (self-)disciplined | Statements about being (self-)disciplined and following through with a plan. | “Konsequent zu arbeiten und mich in der Früh auch wirklich an den Schreibtisch zu sitzen und etwas zu machen.“ |  |
| *3.5. Being organized* |  |  | |
| 3.5.1. Being organized in general | Statement referring to successfully structuring and organizing oneself in general.  Statements about successfully 🡪 3.5.1. Successfully upholding a daily structure, 🡪 3.5.2. Successfully managing tasks and time, 🡪 3.5.3. Successfully keeping track of tasks to be done 🡪 3.5.4. Successfully adhering to deadlines were coded in the respective subcategory. | “Die Organisation” | |
| 3.5.2. Successfully upholding a daily structure | Statements about successfully keeping up a daily routine or structure. | „Meine Routine einzuhalten“ | |
| 3.5.3. Successfully managing tasks and and time | Statements about successfully managing time and/or creating a learning plan. Also, managing to plan in breaks. | „Die Einteilung, wann ich was mache“ | |
| 3.5.4. Successfully keeping track of tasks to be done | Statements about successfully keeping track of the tasks to be done. | „habe einen guten Überblick über den derzeitigen Stoff“ | |
| 3.5.5. Successfully adhering to deadlines | Statements about successfully adhering to deadlines. | „Die Arbeitsaufträge pünktlich abzugeben.“ | |
| **4. Contextual conditions** |  |  | |
| *4.1. Having good learning materials* | Statements about having good learning materials to work with. | „Das die Unterlagen gut vorbereitet sind.“ | |
| *4.2. Good learning environment* | Statements about having a quieter and less distracting learning environment at home. | „Das ich alleine bin und mich nicht immer wer stört“ | |
| *4.3. Successful digital learning* |  |  | |
| 4.3.1. Successful digitally mediated learning | Statements about successful online learning. | „dass ich viele online Übungen machen kann oder manches gut auf YouTube erklärt wird“ | |
| 4.3.2. Successful online communication |  |  | |
| 4.3.1.1. Receiving and handing in assignments & online assignments | Statements about successfully receiving or handing in assignment or doing online assignments. | „Aufgaben die ich am Computer machen kann“ | |
| 4.3.1.2. Successfully working with communication platforms | Statements about successfully working with communication platforms. | „ich kann gut mit den Online-Plattformen umgehen und komme so immer schnell an mein Zeug.“ | |
| 4.3.3. Successfully working on the computer |  |  | |
| 4.3.3.1. Successfully working on the computer in general | Statements about (successfully) working on the computer/getting better in dealing with technical equipment. | „kann immer besser mit dem Computer umgehen.“ | |
| 4.3.3.2. Advantages when working at the computer | Statements about advantages when working with the computer. | „Texte am Computer zu schreiben, weil man viel leichter Sachen ändern kann.“ | |
| 4.3.3.3. Liking to work on the computer | Statements about enjoying working on the computer. | „Ich tue mir leichter bei Home-Office, weil ich es liebe mit dem Computer zu arbeiten.“ | |
| 4.3.4. Having good equipment | Statements about having good or better technical equipment in home-learning than in school. | „da ich an meinem eigenen Rechner (PC) arbeiten kann, welcher Daten und anders in meinem Fall schneller verarbeiten kann, als ein Schul-PC.“ | |
| **5. Well-being** |  |  | |
| *5.1. Successfully keeping up psychological well-being* | Statements about being less stressed and feeling less pressured, taking breaks when necessary and having a better learn-live balance. | „kein Stress, kein Druck“ | |
| *5.2. Successfully keeping up physical well-being* | Statements referring to the situation being physically challenging (e.g. not enough time or space to work out). | „Essen (ich hab das erste mal seit den Sommerferien wieder bewusst gegessen und zwar etwas gesundes, kein Fastfood oder so...“ | |
| **6. Everything is going well** | Statements that everything is going well at the moment. | „Alles“ | |
| **7. Nothing is going well** | Statements that nothing is going well right now. | „nichts gelingt besser als während der Schulzeit“ | |
| **8. Residual Category** | Non-content-bearing statements, non-topic-related answers, answers with insufficient specification | „Alles so wie in der Schule.“ | |
