# Supplementary material for: Learning during COVID-19: the role of self-regulated learning, motivation, and procrastination for perceived competence
Source: Z Erziehwiss. 2021 Mar 4;24(2):393–418. doi: 10.1007/s11618-021-01002-x (PMC7931168; doi:10.1007/s11618-021-01002-x)
Supplement: Supplementary file 6 — Table VI. Category system for Question 3: “With what could you currently use some help?” [file 11618_2021_1002_MOESM6_ESM.docx]

## Table VI

## *Category system for Question 3: “With what could you currently use some help?”*

| **Category** | | **Coding rules** | | **Examples** | |
| --- | --- | --- | --- | --- | --- |
| **1. Contact with others** | |  | |  | |
| *1.1. Wanting contact with/support from guardians/family* | | Statements about wanting or needing contact with or support from family or caregivers. | | „es wäre toll wenn meine Mutter mich unterstützen würde und mich motivieren würde wenn ich mal nicht so viel schaffe.“ | |
| *1.2 Wanting contact with/support from peers* | | Statements about wanting more contact or with other students and/or opportunities for learning in groups. | | „Teamarbeit mit Kollegen“ | |
| *1.3. Wanting contact with/support from teachers* | |  | |  | |
| 1.3.1. Wanting contact with/support from teachers in general | | Statements that more contact with teachers is wanted in general. Also expressing the need for synchronized communication (e.g. video conferences). | | „mehr persönliche Betreuung durch Lehrer/Videochats würden helfen (manche Lehrer bieten es aber nicht an)“ | |
| 1.3.2. Needing clear instructions/help understanding assignments | | Statements about wanting clear instructions or need help understanding assignments. | | „Die Aufgaben zu verstehen da die Lehrer nur in Kurzfassung hin schreiben was man tun soll.“ | |
| 1.3.3. Wanting a teacher to explain things | | Statements referring to the wish of more explanation from teachers. | | „Dass mir der Lehrer die Sachen erklärt.“ | |
| 1.3.4. Wanting the opportunity to ask questions | | Statements about needing the opportunity to ask questions or about getting timely answers. | | „Bei gewissen Fragen, die ich selber nicht beantworten kann.“ | |
| 1.3.5. Wanting (timely) feedback | | Statements expressing the wish for getting (timely) feedback. | | “Möglichst rasche Rückmeldung der Lehrer“ | |
| 1.3.6. Wanting teachers to have empathy/understanding for the situation | | Statements about how teachers don’t show empathy and that understanding of the situation would be needed. | | „Ich frage mich warum ich überhaupt noch versuche mitzukommen, da es den Lehrern scheinbar eh egal ist.“ | |
| **2. Learning outcomes** | |  | |  | |
| *2.1. Wanting support in completing assignments* | | Statements about wanting support in completing assignments (also working faster and more productively as well as more diligently and accurately). | | „vorallem schneller mit dem erledigen der Aufgaben zu werden“ | |
| *2.2. Wanting support in learning and/or understanding (new) material* | | Statements about wanting support in learning or comprehending new or complicated material or in rehearsing old material.  Statements referring to support in independently learning (new) material were also coded under 🡪 3.2. Wanting support in learning alone/independently.  Statements referring to the need for teachers to explain (new) material were also coded in 🡪 1.3.3. Having a teacher explaining things  Statements about needing support in learning (new) material in a specific subject were also coded in 🡪 2.4. Wanting support with specific subjects/tasks/assignments in the respective subcategory. | | „Bei neuem Stoff.” | |
| *2.3. Wanting support in preparing for the final exams (Matura)* | | Statements about preparation for the final exams (Matura) being challenging. | | „Matura Vorbereitung“ | |
| *2.4. Wanting support in/with specific subjects/tasks/assignments* | | Statements about needing support referring to specific subjects, final exams or subjects, that are perceived as boring or unimportant are coded in the respective subcategory (subjects that were mentioned more than 20 times were kept in separate categories). | |  | |
| 2.4.1. Wanting support in/with specific subjects/tasks/assignment in general | | Statements about needing support in tasks, assignments or activities related to school or learning that are perceived as particularly difficult. | | „Beim Erklären der neuen Grammatiken in den verschiedenen Fächer.“ | |
| 2.4.2. Wanting support in working for (perceived) boring/unimportant subjects | | Statements about needing support in subjects that are perceived as boring, unimportant. | | „Bei den Gegenständen oder Aufgaben die meiner meinung nach nicht notwenig sind“ | |
| 2.4.3. Wanting support in Mathematics | | Statements about needing support in Mathematics.  Statements referring to the need of support in learning or understanding (new) material in Mathematics are also coded in 🡪 2.2. Wanting support in learning and/or understanding (new) material.  Statements referring to needing explanations from teachers in Mathematics are also coded in 🡪 1.3.3. Having a teacher explain things. | | „Nur in Mathematik.“ | |
| 2.4.4. Wanting support in German | | Statements about needing support in German.  Statements referring to the need of support in learning or understanding (new) material in German are also coded in 🡪 2.2. Wanting support in learning and/or understanding (new) material.  Statements referring to needing explanations from teachers in German are also coded in 🡪 1.3.3. Having a teacher explain things. | | „Beim Geschichten schreiben in Deutsch.” | |
| 2.4.5. Wanting support in English | | Statements about needing support in English.  Statements referring to the need of support in learning or understanding (new) material in English are also coded in 🡪 2.2. Wanting support in learning and/or understanding (new) material.  Statements referring to needing explanations from teachers in English are also coded in 🡪 1.3.3. Having a teacher explain things. | | „Englisch” | |
| 2.4.6. Wanting support in Physics | | Statements about needing support in Physics.  Statements referring to the need of support in learning or understanding (new) material in Physics are also coded in 🡪 2.2. Wanting support in learning and/or understanding (new) material.  Statements referring to needing explanations from teachers in Physics are also coded in 🡪 1.3.3. Having a teacher explain things. | | „beim Physik Stoff” | |
| 2.4.7. Wanting support in economical subjects | | Statements about needing support in economical subjects.  Statements referring to the need of support in learning or understanding (new) material in economical subjects are also coded in 🡪 2.2. Wanting support in learning and/or understanding (new) material.  Statements referring to needing explanations from teachers in economical subjects are also coded in 🡪 1.3.3. Having a teacher explain things. | | „Buchhaltung”  „Rechnungswesen” | |
| 2.4.8. Wanting support in other subjects | | Statements about other subjects being challenging (e.g. Latin, Music, Chemistry, ...).  Statements referring to the need of support in learning or understanding (new) material in other subjects are also coded in 🡪 2.2. Wanting support in learning and/or understanding (new) material.  Statements referring to needing explanations from teachers in other subjects are also coded in 🡪 1.3.3. Having a teacher explain things. | | „Spanisch” | |
| **3. Learning process** | |  | |  | |
| *3.1. Wanting support in the learning process in general* | | Statements about needing support with learning in general (e.g. not knowing how to learn). | | „wie ich lernen soll” | |
| *3.2. Wanting support in learning alone/independently* | | Statements about needing support in learning to learn independently.  Statements referring needing support from others were coded in 🡪 1. Wanting contact with/support from guardians/family and in the respective subcategories. | | „Ich muss lernen, wie ich mir selbst Sachen beibringen kann.” | |
| *3.3. Wanting support in learning to concentrate/avoid distractions* | | Statements about needing help to concentrate for longer periods and avoid distractions.  Statements referring to needing a quieter and less distracting learning were coded in 🡪 4.2. Wishing for a supportive learning environment. | | „Das ich mich sehr viel besser konzentrieren kann.“  „ich bräuchte Hilfe Konzentration zu behalten um sie dann auch richtig einzusetzen“ | |
| *3.4. Wanting support with motivational and volitional challenges* | |  | |  | |
| 3.4.1. Wanting support in building/maintaining motivation | | Statements about wanting support in building and maintaining motivation and/or getting started with tasks. | | „Ich brauche jemanden, der mich motiviert“ | |
| 3.4.2. Wanting support in building/maintaining (self-)discipline | | Statements about wanting support in building and maintaining (self-)discipline (e.g. following through with a plan). | | „Ich bräuchte jemanden, der mich kontrolliert, damit ich nicht abschweife.“ | |
| *3.5. Wanting support with (self-) organization* | |  | |  | |
| 3.5.1. Wanting support with (self-) organization in general | | Statements referring to wanting support keeping organized and structured.  Statements about wanting 🡪 3.5.2. Wanting support in building/maintaining a daily structure, 🡪 3.5.3. Wanting support in managing tasks and time, 🡪 3.5.4. Wanting support in keeping track of tasks to be done and 🡪 3.5.5. Wanting support in adhering to deadlines were coded in the respective subcategory. | | „Teilweise bei der Organisation“ | |
| 3.5.2. Wanting support in building/maintaining a daily structure | | Statements referring to wanting support in keeping up a daily routine or structure. | | „Beim Strukturieren vom Alltag“ | |
| 3.5.3. Wanting support in managing tasks and time | | Statements about wanting support in managing time well and/or creating a learning plan. | | „Einteilen der Arbeitsaufträge.“ | |
| 3.5.4. Wanting support in keeping track of tasks to be done | | Statements about wanting support in keeping track of the tasks to be done.  Statements about wanting support adhering to deadlines due to keeping track of different deadlines was also coded in 🡪 3.5.5. Wanting support in adhering to deadlines.  Statements about wanting a unification of communication platforms were coded in 🡪 4.4.1. Wishing for teachers to be more tech savvy | | „Beim behalten der Übersicht“ | |
| 3.5.5. Wanting support in adhering to deadlines | | Statements about wanting support adhering to deadlines. | | „Deadlines einhalten“ | |
| **4. Contextual conditions** | |  | |  | |
| *4.1. Wanting good learning materials* | | Statements regarding the need for good learning materials. | | „Bessere Material Bereitstellung der Lehrer“ | |
| *4.2. Wishing for a supportive learning environment* | | Statements that students would like to have a conductive (e.g. calm) learning environment. | | „Ein eigener Raum nur fürs Lernen wäre super.“ | |
| *4.3. Wanting support in dealing with school-related requirements* | |  | |  | |
| 4.3.1. Wishing for support in dealing with too high school-related requirements | |  | |  | |
| 4.3.1.1. Wishing for better agreement/coordination between the teachers | | Statements about wishing that teachers would coordinate their assignments.  Statements regarding the wish for fewer communication platforms were coded in 🡪 4.4.4.1. Wishing for unified communication | | „Absprache der Lehrer untereinander“ | |
| 4.3.1.2. Wishing for more time for finishing assignments | | Statements about wanting more time for finishing assignments. | | „mehr Zeit für Aufgaben“ | |
| 4.3.1.3. Wishing for fewer assignments | | Statements about wanting less time and work demanding assignments. | | „Ich würde mir nur wünschen, dass die Lehrer sich ein bisschen zurückhalten mit den Arbeitsaufträgen.“ | |
| 4.3.2. Wishing for more learning material to further interest/talents | | Statements about wishing for more difficult learning materials to further interest and/or talents regular subjects. | | „Beim gezielten Vorbereiten auf naturwissenschaftliche Wettbewerbe, da diese Themengebiete umfassen, die nicht Teil des Schulstoffes sind“ | |
| *4.4. Wishing for support in dealing with the distance learning setting* | | Statements about needing support with distance learning or wishing for regular lessons. | | „Ich würde gerne wieder mehr analog lernen, digitales Lernen ist konzentrationsbedingt sehr anstrengend“ | |
| 4.4.1. Wishing for teachers to be more tech savvy | | Statements about wishing that teachers were more tech savvy. | | „Dabei Lehrer ins Jahrhundert der Technologie zu bringen.“ | |
| 4.4.2. Wishing for assistance in receiving/handing in/doing (online) assignments | | Statements about needing support with receiving or handing in assignments and doing online assignments. | | „Eine Einheitliche Plattform mit allen Arbeitsaufträgen gesammelt.“ | |
| 4.4.3. Wanting support in digitally mediated teaching & learning | | Statements about needing support in learning from digital media or when material is taught digitally. | | „der neue Stoff nur über den Computer zu lernen ist mühsam“ | |
| 4.4.4. Wanting support in working with communication platforms | |  | |  | |
| 4.4.4.1. Wishing for unified communication | | Statements about the desire for teachers to use fewer communication platforms and coordinate their ways of communication. | | „Das jeweilige Professoren nicht über eine Platform kommunizieren, sondern über mehrere.“ | |
| 4.4.4.2. Wanting support when communication platforms don’t work | | Statements about needing support when the platforms used are not working. | | „Teils bei den Online Programmen, da sie nicht funktionieren oder überlastet sind.“ | |
| 4.4.5. Wanting support in working on the computer | | Statements about needing support in dealing with the computer (software or hardware) or doing research on the internet. | | „wenn man etwas im internet suchen muss“ | |
| 4.4.6. Wanting support in dealing with technical equipment or requirements | | Statements about needing support when dealing with technical equipment in context of distance learning. | | „Computerprobleme“ | |
| 4.4.6.1. Needing the necessary equipment | | Statements about wishing for sufficient technical equipment. | | „Vielleicht einen eigenen Laptop?“ | |
| 4.4.6.2. Needing a better internet connection/(wireless) network | | Statements about needing a better internet connection and/or (wireless) connection. | | „Besseres und stabiles Internet“ | |
| **5. Well-being** | | Statements about wanting help in dealing with stress and aggression or psychological well-being in general. | | „Psychische Unterstützung wäre nicht schlecht“ | |
| **6. Support is needed in everything** | | Statements that there is a need for support (almost) everywhere. | | „überall“ | |
| **7. No further support necessary** | | Statements that there is no need for support, either because students can deal on their own or already get enough support.  In statements that there is no help needed APART from (...), the statements was split. The part where there was stated that no help is needed was coded in the 🡪 8. Residual category, and the part where need for support was expressed was coded in the respective category. | | „Bei gar nichts.“ | |
| **8. Residual Category** | | Non-content-bearing statements, non-topic-related answers, answers with insufficient specification | | „Keine ahnung“ | |
